# Cocreating Principles for Digital Health Equity: Cross-Sectional, Qualitative Study for Participatory Human-Centered Design in Catalonia

**DOI:** 10.2196/84129

**Published:** 2026-01-06

**Authors:** Jordi Piera-Jiménez, Núria Vilarasau Creus, Ada Maymó Costa, Xabier Michelena, Andrea Climent Fageda, Alèxia Farré, László Herczeg, Lekshmy Parameswaran, Gerard Carot-Sans, Luis Valle

**Affiliations:** 1 Digitalization for the Sustainability of the Healthcare System (DS3) research group, Barcelona, Spain; 2 Information Sysems Directorate, Catalan Health Service, Gran Via de les Corts Catalanes 587, Barcelona, 08007, Spain, 34 651041515; 3 The Care Lab, Barcelona, Spain; 4 Rheumatology Research Group, Vall d’Hebron Research Institute, Barcelona, Spain

**Keywords:** digital health, equity, human-centred design, participatory design, digital health strategy, health information systems, co-creation, user experience

## Abstract

**Background:**

Digital health technologies promise to democratize health care access yet often exacerbate existing inequalities when developed through traditional top-down approaches that prioritize technology implementation and exclude end users from design processes. The COVID-19 pandemic accelerated digital transformation while simultaneously exposing how technology can both bridge and widen gaps in health care access. Understanding how to systematically integrate equity considerations into digital health transformation across entire health systems has become increasingly urgent.

**Objective:**

This study aims to cocreate actionable design principles for equitable digital health transformation through a large-scale participatory human-centered design (PHCD) process involving diverse stakeholders across Catalonia’s health care ecosystem (northeast Spain), with the aim of establishing guidelines for information systems that support a person-centered, integrated, and longitudinal care delivery model.

**Methods:**

We conducted a qualitative PHCD research study involving 265 participants representing diverse stakeholder groups: citizens and informal caregivers (n=106), health care professionals (n=83), health care managers and leaders (n=50), and experts representing various domains of digital health innovation (n=26). Through two sequential rounds of participation between June 2024 and April 2025, we used design thinking methodologies and cocreation tools in 24 sessions across Catalan geography and 7 topic-specific expert sessions. Data collection used innovative visual tools, including journey mapping, care model animations, future scenario storyboarding, and facilitated ideation techniques. Analysis followed an inductive-deductive approach combining affinity mapping, thematic synthesis, and participant validation to transform stakeholder proposals into actionable design principles.

**Results:**

Participants identified critical barriers to digital health equity, including digital literacy gaps, fragmented information systems, a lack of user involvement in design, and insufficient consideration of vulnerable populations' needs. The cocreation process yielded 10 fundamental principles: (1) the person and their care circle at the center, (2) health for everyone, everywhere, (3) tools for more compassionate care, (4) a better professional experience, (5) an active role of the population, (6) personalized and proactive care, (7) systematic use of data for decision-making, (8) integrated quality data working for health, (9) an information system that builds trust, and (10) collaboration as a driver of innovation.

**Conclusions:**

This study shows how large-scale, rigorously conducted PHCD can uncover and address equity barriers in health information systems. Beyond producing 10 actionable design principles, it highlights how engaging diverse stakeholders can turn complex inequities into practical guidance for equitable digital transformation. The resulting principles provide a framework for creating person-centered systems that are robust, inclusive, and accessible to all, while underscoring the need for enduring partnerships among public institutions, researchers, design experts, and communities as a foundation for sustainable and equitable digital health innovation.

## Introduction

The promise of digital health technologies to transform health care delivery has never been more apparent than in the wake of the COVID-19 pandemic. While the rapid adoption of telemedicine and mobile health apps demonstrated the potential of technology to maintain health care access during unprecedented disruption, it simultaneously revealed a troubling paradox: the very innovations designed to improve health care access often exacerbated existing inequities [1,2]. Vulnerable populations, including older adults, those with limited digital literacy, people with disabilities, and communities facing language barriers, frequently found themselves excluded from these digital advances, transforming potential bridges to care into barriers [3,4].

This paradox stems from a fundamental flaw in how health technologies are traditionally developed. Despite decades of evidence supporting user-centered approaches, health information systems continue to be designed primarily through top-down processes that prioritize technical requirements and organizational workflows over the needs, contexts, and capabilities of end users [5-7]. Health care professionals report spending up to 50% of their time on administrative tasks related to electronic health records (EHRs), reducing time available for direct patient care [8], while at the same time, patients struggle to navigate increasingly complex digital interfaces that assume levels of digital literacy many do not possess [9]. These challenges are particularly pronounced for vulnerable populations whose voices are rarely heard in technology design processes [10].

Participatory human-centered design (PHCD) offers a radically different approach to health technology development. Rooted in the principle that those who will use a system should be central to its design, PHCD engages users as partners throughout the development process, from initial problem definition through implementation and evaluation [11-14]. In health care contexts, this means involving patients, caregivers, health care professionals, and community members not merely as sources of requirements but as cocreators of solutions [15,16]. This orientation resonates with the broader global health literature describing human-centered design as a disciplined yet flexible practice that bridges design and implementation by iteratively engaging stakeholders and aligning innovation with human values and equity goals [17,18]. This approach is particularly crucial for addressing health equity, as it ensures that the voices of those most at risk of digital exclusion, often the same populations experiencing the greatest health disparities, are not just heard but actively shape the solutions developed [19,20]. Growing evidence demonstrates that health information systems developed through genuine participatory processes achieve higher adoption rates, better user satisfaction, and more equitable outcomes across diverse populations [21]. Yet, recent reviews highlight that while human-centered approaches can enhance equity for participants directly involved in cocreation, their broader, system-level impact often remains untested due to limited scaling and evaluation [22].

The concept of digital health equity extends far beyond mere access to technology. As defined by the World Health Organization (WHO), it encompasses the ability of all individuals to benefit from digital health solutions regardless of their socioeconomic status, geographic location, age, disability, or cultural background [23]. Operationalizing this vision requires addressing what recent collaborative frameworks identify as digital determinants of health (i.e., the complex, interconnected factors that shape whether digital health interventions produce equitable outcomes across populations) [24-27]. The adaptation of Richardson et al [26] of the National Institute of Minority Health and Health Disparities Research Framework [28] suggests that digital health equity operates across 4 different levels of influence (individual, interpersonal, community, and societal), each containing specific determinants that must be simultaneously addressed [26]. Similarly, recent work on equitable digital health design further proposes operational frameworks (eg, the Double Diamond and IDEAS models) that guide practitioners in deliberately embedding equity considerations throughout all design phases and in fostering structured collaboration with underserved groups [29].

Like many other countries worldwide, the COVID-19 crisis accelerated the digital transformation of health care in Catalonia (northeast Spain), with digital consultations increasing from 2% to more than 25% of all primary care visits between 2019 and 2021 [30,31]. However, this rapid digitalization revealed significant disparities across the region’s 8 million inhabitants of the region. Rural areas struggled with inadequate internet infrastructure, older adult populations faced overwhelming digital interfaces, immigrant communities encountered language barriers in digital platforms, and health care professionals grappled with fragmented systems that hindered rather than helped care coordination [32]. These challenges acquired particular significance as Catalonia prepared to implement its ambitious Digital Health Strategy (2026‐2031), a transformative initiative designed to support the future needs of health care delivery. Before embarking on this large-scale transformation, policymakers determined that equity principles must be embedded from the outset to avoid amplifying existing disparities.

Recognizing both the urgent need to address these digital equity challenges and the imperative to build equity into the upcoming Digital Health Strategy from its foundation, the Catalan Department of Health launched on a major participatory initiative to reimagine health information systems for the future. Rather than starting with predetermined technological solutions, the Department adopted a PHCD approach that would first identify gaps in the current health care delivery model and envision how future care should be organized, then determine how health information technologies could support this transformation. Following this approach, the idea was to reflect the growing recognition that creating truly equitable health information systems requires fundamentally rethinking not just what we build, but how we build it and who is involved in the building process [33].

This paper presents the results of the aforementioned large-scale PHCD process conducted across Catalonia, aimed at providing guidance to the design of a new and equitable health information infrastructure that places the citizen at the center. The study addressed 2 main questions: (1) What are the lived experiences and challenges of diverse stakeholders navigating the Catalan health care system, particularly regarding barriers to equitable access and digital health engagement? (2) What fundamental principles and guidelines should inform the design of health information systems to ensure they are equitable, accessible, and responsive to the needs of all populations, especially those traditionally excluded from design processes?

Beyond these specific research questions, the study constitutes the first large-scale use case of user involvement in human-centered design for health care system transformation, providing valuable insights for replicating and scaling participatory design approaches across complex health systems.

## Methods

### Study Design and Conceptual Framework

We conducted a qualitative participatory design research study to develop a collective vision for Catalonia’s future health care model and actionable design principles for digital health information systems. Our framework combined 2 complementary approaches. Human-centered design placed people at the core of the process, grounding design in their needs and contexts [34,35]. Participatory design ensured that diverse stakeholders were active cocreators rather than passive consultees [36]. This combined approach (ie, PHCD) was chosen because it directly addresses a fundamental cause of digital health inequity: traditional top-down development processes that systematically exclude the voices of those most affected by health disparities, particularly vulnerable populations who experience the greatest barriers to digital health access. PHCD engages stakeholders at all levels, from citizens and caregivers to frontline professionals, managers, and technical experts, capturing how barriers manifest and interact across individual, interpersonal, organizational, and systemic dimensions. This approach ensures a deep understanding of user needs before developing solutions, avoiding the premature solutioning that often leads to health technology failure [11,37].

The study design was structured around 2 sequential phases of participation, each addressing distinct but interconnected research questions. Both rounds used consistent participatory design methods, including journey mapping, system visualization, facilitated ideation, and collaborative prioritization, with Round 1 tools focusing on current care experiences and future care model visioning, and Round 2 tools adapted to translate identified needs into specific design principles for health information systems. Round 1 was deliberately designed to focus on health care delivery experiences and needs without introducing technology considerations, thereby avoiding the risk of biasing discussions toward predetermined technical solutions or constraining participant imagination to existing digital tools. This approach ensured that identified barriers and desired futures emerged authentically from lived experiences rather than being shaped by assumptions about technological feasibility or current system constraints. Only after establishing this grounded understanding of real citizen and professional needs did Round 2 shift focus to cocreating specific design principles for health information systems, ensuring that technology would serve human needs rather than drive the transformation agenda. The 2-phase approach enabled progressive deepening of understanding, moving from broad exploration of health care system experiences to focused cocreation of specific design principles for future health information systems. Finally, we deliberately adopted a multilevel scope encompassing technological design, organizational implementation, and broader system considerations. This approach was grounded on the fact that digital health equity failures occur across all these dimensions [26,38-40], and our PHCD methodology required understanding the full health care delivery context before narrowing to specific IT requirements.

The manuscript has been prepared according to the Standards for Reporting Qualitative Research (SRQR) guidelines [41].

### Project Team Organization

The study was managed through a collaborative governance structure designed to ensure scientific rigor, meaningful stakeholder engagement, and operational excellence. A project steering board comprised representatives from the Catalan Health Service (JP-J, XM, LLV, and AC). The steering board was responsible for defining project objectives, monitoring progress against milestones, ensuring alignment with regional health transformation goals, making decisions about methodological adaptations based on emerging insights, and engaging with territorial health coordinators to recruit participants.

The operational implementation was carried out by a specialized design practice in multistakeholder engagement, human-centered design, and participatory design methodologies for enabling health and care system transformation. This team included supervisors (LP and LH) who provided methodological guidance and oversaw the overall design process to ensure consistency and quality. A dedicated tandem project management support structure coordinated logistics, including scheduling meetings across multiple territories, managing participant communications, providing technical support, and coordinating the overall design methodology and facilitation team (NVC). A team of 4 trained service-system designers who designed, facilitated, and synthesized the sessions, 2 lead facilitators who guided overall discussion flow and ensured all voices were heard (NVC and AMC), and 2 support facilitators who helped to document information during sessions, assisted participants with cocreation activities, and provided real-time analysis support (AF and AC). This team structure ensured comprehensive documentation while enabling smooth session flow and participant engagement.

The steering board and operational team established a coordinated governance structure with weekly alignment sessions to ensure methodological consistency, address implementation challenges, and maintain project objectives throughout the process.

### Participants and Recruitment

Participants' recruitment and involvement adhered to the 4 core criteria outlined in the Catalan Department of Health’s Framework for Citizen Participation in Health, ensuring adequate methodological definition, maintaining scientific evidence and ethical standards, protecting vulnerable and minority populations, and preserving public health system sustainability [42].

Recruitment followed a purposive sampling strategy designed to maximize diversity and ensure representation of voices often excluded from technology design processes [43]. Sampling was operationalized through the health coordinators of each Catalan health region. These coordinators, who are managers within the Catalan Health Service, are responsible for planning and translating system-level health care policies into practice across the 10 health care regions of Catalonia. Once the study protocol was approved, and before fieldwork started, all 10 health coordinators attended a kick-off meeting in which the research team (ie, the authors) presented the study aims and procedures and provided detailed instructions for participant identification and recruitment. Comprehensive descriptions of the selection criteria and recruitment process are provided in the Multimedia Appendix 1. The health coordinators identified potential participants based on explicit diversity criteria, including gender and age distribution, organization types (public, private, and nonprofit sectors), health and social care service levels, and geographic representation across urban, semiurban, and rural territories. Health coordinators were explicitly asked to include, whenever possible and while meeting eligibility requirements, participants from potentially vulnerable groups such as older adults, immigrants, individuals with disabilities, and those with lower socioeconomic status. The project manager conducted weekly meetings with health coordinators for monitoring the recruitment process.

The study targeted four key stakeholder groups, each bringing essential perspectives to the cocreation process: (1) citizens and informal caregivers with diverse health care experiences (including expert patients and representatives of patient associations), who provide lived experience of navigating the health care system and represent the end user perspective; (2) health and social care professionals across different disciplines and care settings, who offer frontline insights into system functionality and daily operational challenges; (3) health and social care managers and leaders from various organizational levels, who contribute strategic and organizational perspectives on system implementation and sustainability; and (4) experts representing various domains of strategy, innovation, and technology, who provide technical expertise and broader sectoral knowledge to inform feasible and evidence-based solutions.

### Territorial and Contextual Considerations

We recognized that territorial context significantly influences health care experiences and digital health needs [44,45]. Our sampling strategy deliberately included representation across urban, semiurban, and rural territories to ensure diverse contextual factors would be captured [3]. We also considered intersectional factors in our analysis framework, recognizing that age, gender, socioeconomic status, immigration status, and disability interact with geographic location to create unique barriers and opportunities [10]. We conducted an informed selection of the locations in consultation with the respective health regions to ensure comprehensive coverage of key regions and strategic areas across the Catalan territory.

### Timeline and Methods

**Figure 1.**
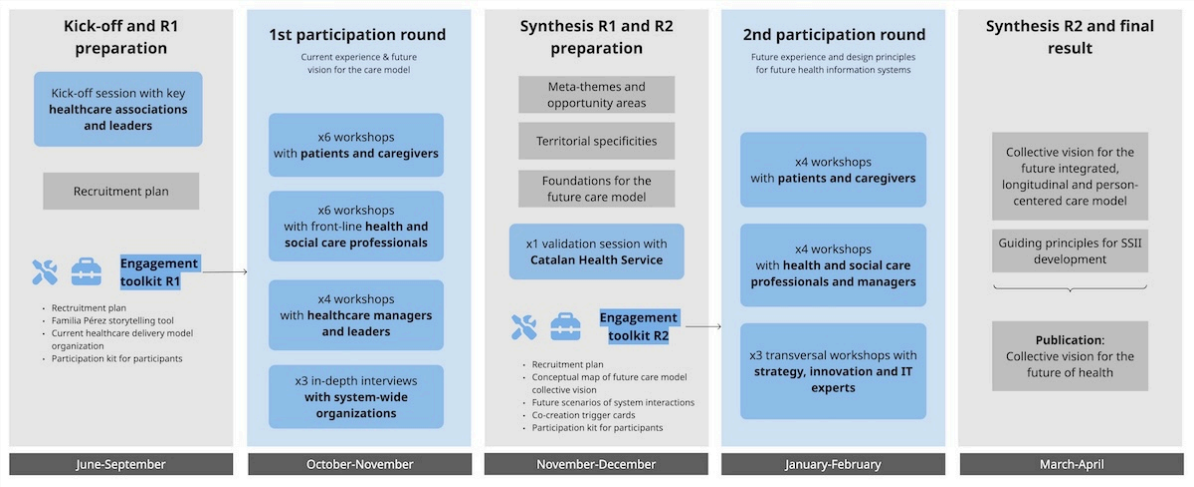
Project timeline, with activities (in blue) and outputs (in gray). R1: round 1; R2: round 2.

The study spanned from June 2024 to April 2025, beginning with preparation activities conducted from June to September 2024 (Figure 1). Data collection occurred through 2 sequential rounds of participation and analysis from October 2024 to March 2025, followed by consolidation of results and synthesis conducted from March to April 2025. Each round addressed distinct but interconnected research objectives, building progressively from understanding current health care experiences to cocreating specific design principles for future digital health information systems.

Round 1, conducted from October to November 2024, focused on collective reflection about the care model. This round aimed to understand current lived experiences with the health care system, identify what works well and what needs improvement, and envision desired futures. The central guiding questions included, “What do we understand by a person-centered, longitudinal, and integrated care model?” “What needs does this model address?” and “What must change in the current system to achieve this vision?” Data were collected throughout 16 cocreation sessions conducted face-to-face and strategically selected to represent Catalonia’s geographic and demographic diversity. Sessions were distributed across urban territories (Badalona, Barcelona, Girona, Lleida, and Tarragona), semiurban territories (Igualada, Manresa, Sant Cugat, Sant Feliu de Llobregat, Tortosa, and Vic), and rural territories (Amposta, Navàs, Palamós, Tremp, and Sort). Each 2-hour session included between 8 and 12 participants and followed a carefully structured format designed to maximize participation and insight generation. Additionally, 3 online one-hour group interviews with 2 participants each complemented the sessions across the territory, focusing on macrolevel perspectives and strategic insights into system-level challenges and opportunities. The organizations participating in these interviews were: the Agency for Quality and Assessment in Healthcare, the TICSalut and Social Foundation, and the Catalan Integrated Care Agency.

Round 2, conducted between January and February 2025, was devoted to cocreating specific design principles for digital health information systems. Building on insights from Round 1, this round transformed needs and barriers into actionable principles. The central question guiding this round was, “What design principles should guide the future development of health information systems to ensure they serve all citizens equitably?” This round started with a validation session with 7 experts from the Catalan Health Service, who reviewed Round 1 learnings and bridged into defining the focus for Round 2. It used 8 additional face-to-face sessions, maintaining geographic representation while focusing on the development of design principles. These sessions were distributed across urban territories (Barcelona), semiurban territories (Olot, Puigcerdà, Reus, Tàrrega, and Vilanova i la Geltrú), and rural territories (Mora d’Ebre, Solsona). Each session lasted 2 hours and included between 8 and 12 participants. Three topic-specific expert sessions brought together experts in specific domains identified from Round 1 learnings. Each session took 2 and a half hours and included 6 expert participants focusing on particular aspects of digital health transformation, such as strategy, innovation, and technology, providing a more macro perspective on the central question.

**Figure 2.**
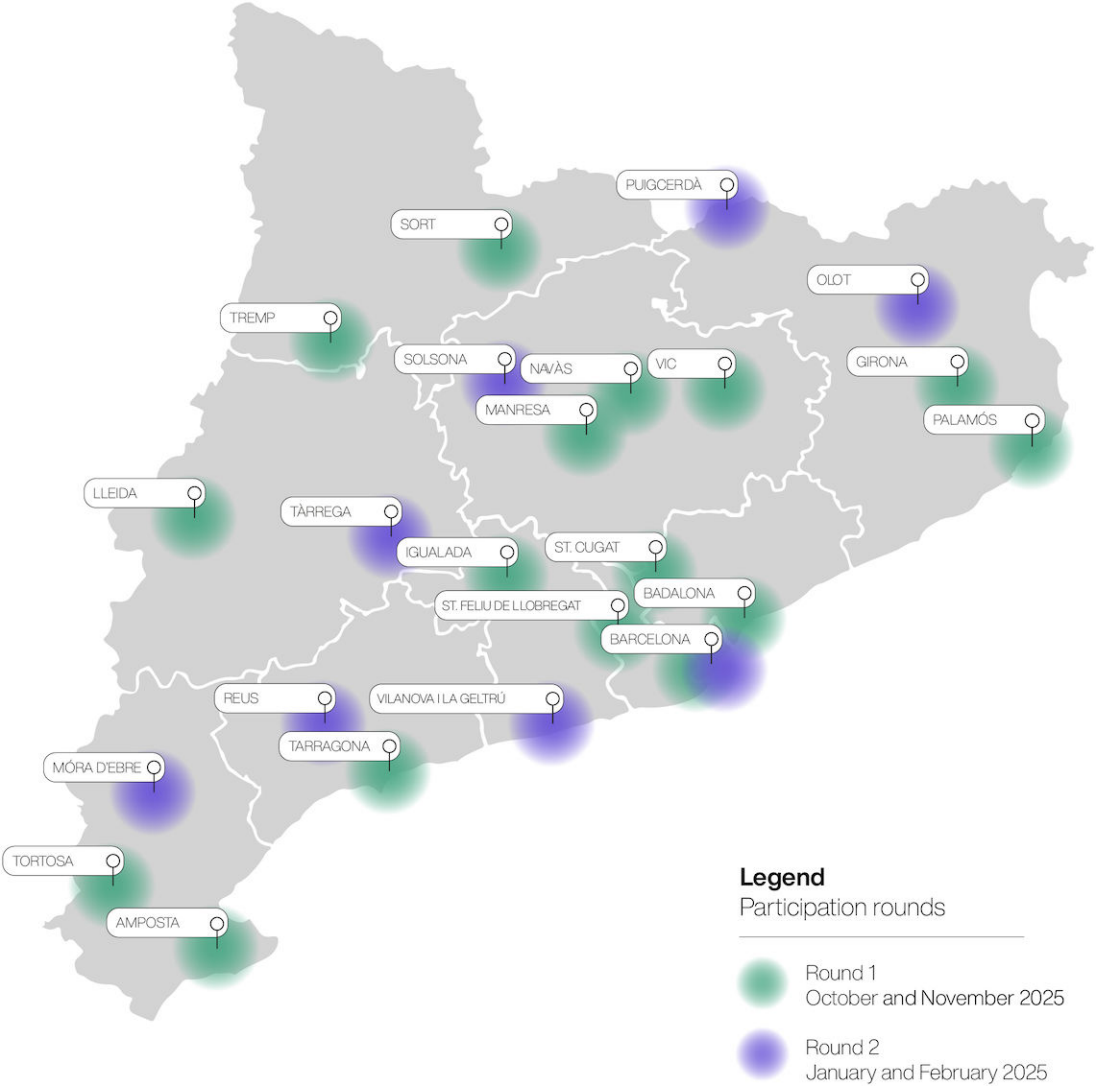
Territorial distribution of the face-to-face participatory sessions across the 2 rounds.

The locations of the 2 rounds across the Catalan geography are depicted in Figure 2.

All sessions followed a consistent structure designed to create safe, inclusive spaces for participation. Sessions began with a 15-minute opening that established context, introduced facilitators, and established participation ground rules to ensure safe sharing of diverse perspectives. Core activities lasting 90‐105 minutes varied by round and session type but consistently used visual facilitation tools, including journey mapping, experience sharing, ideation techniques, and collaborative prioritization exercises. Sessions concluded with 10‐15 minutes for summarizing key insights, outlining next steps, and acknowledging participant contributions.

Sessions used innovative visual tools including journey mapping and storytelling to understand current health care experiences, scenario building for future visioning, and facilitated ideation techniques for developing design principles. Large-format posters, sticky notes, and collaborative boards enabled participants to contribute ideas both verbally and visually (reflecting individually and sharing collectively). Session logistics were carefully adapted to ensure inclusive participation across diverse contexts. Rural sessions addressed infrastructure challenges through flexible scheduling and accessible locations, while urban sessions accommodated participants’ complex schedules while managing larger group dynamics. All venues provided step-free access, appropriate facilities for participants with disabilities, and professional translation services when needed. Transport support was provided where needed, particularly for participants from vulnerable populations or remote areas.

At the end of each session, a satisfaction questionnaire was provided to each participant to assess their participation experience and the perceived value provided by the workshop. The underpinning idea behind this feedback collection was to improve the forthcoming workshop dynamics in case any issue was identified (Multimedia Appendix 1).

### Cocreation Tools

Central to the methodology was the development and use of innovative cocreation tools designed to make participation accessible and engaging for all stakeholders. A total of 4 tools were developed, 2 storytelling visual narratives were designed for the sessions with citizens, caregivers, and professionals. And 2 system maps for the sessions with health care managers and experts. The cocreation materials design drew from established journey mapping [46-48] and system visualization methodologies [49,50] in health care service design. The research team’s approach was informed by their prior development of the WhocCares publication and the WeCare Toolkit (National Council of Social Service, Singapore and fuelfor) for caregiver support in Singapore [51], which demonstrated effective use of visual tools for engaging diverse stakeholders in the cocreation of health and social care service innovations. The full set of tools can be found in the Multimedia Appendix 1.

**Figure 3.**
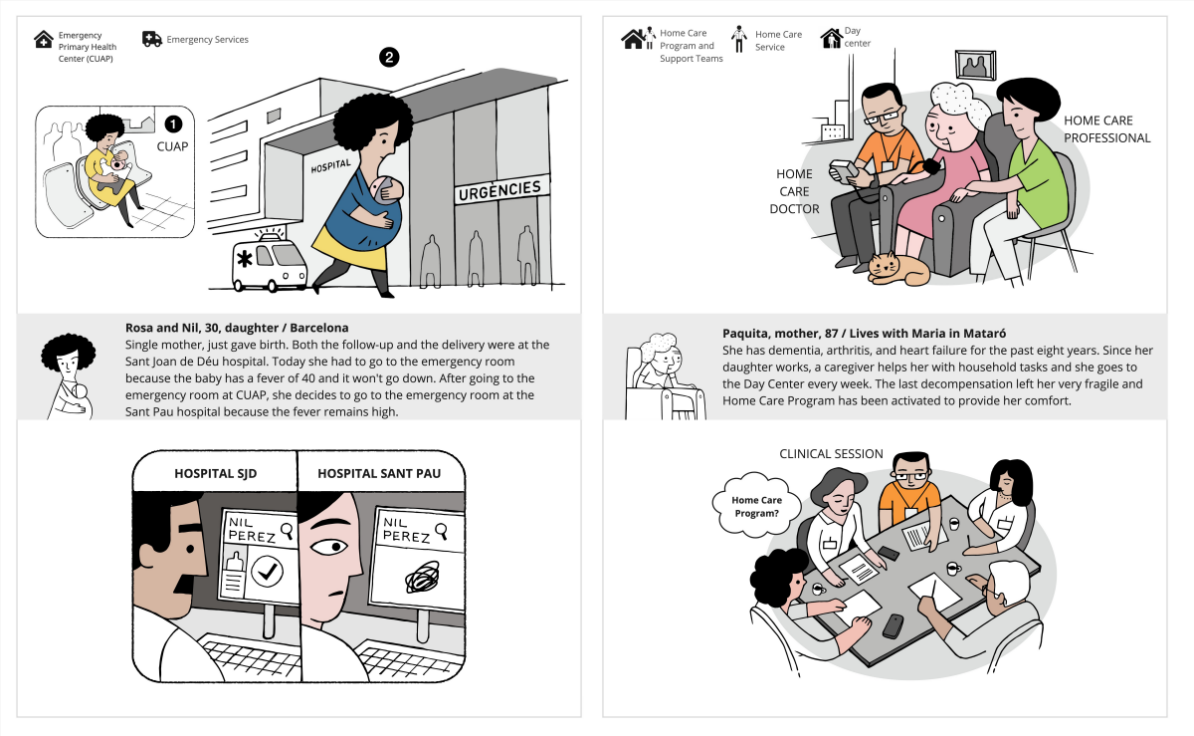
Translated sample of the “Pérez Family” journey mapping and storytelling tool used in face-to-face sessions with patients, caregivers, and professionals.

The “Pérez Family” journey mapping and storytelling tool consisted of a visual narrative depicting 8 health care interaction scenarios across the life course, from prenatal care through end-of-life support (Figure 3). This tool was designed with health care experts from the Catalan Health Service to ensure scenarios were realistic, relatable, and comprehensive, translating complex processes and multiple care pathways into accessible storylines to trigger exploration and discussion. The full poster of the “Pérez Family” tool is provided as supplementary material in Multimedia Appendix 1.

**Figure 4.**
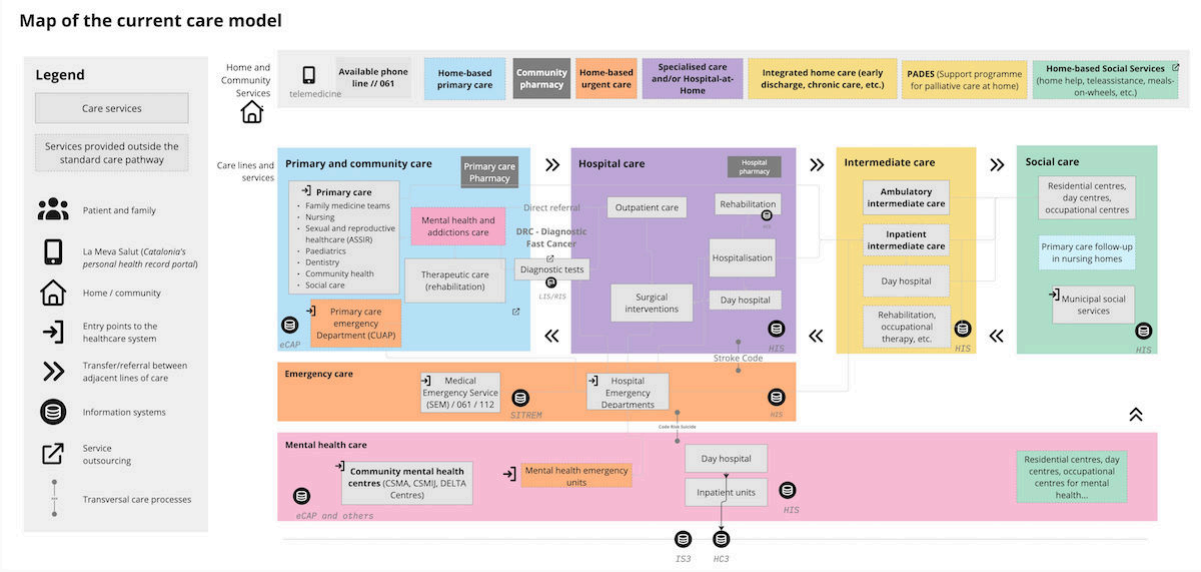
Current health care delivery model organization used in face-to-face sessions with health care leaders and managers. IS3 (*Integrador de serveis de salut*): care process management; HC3 (*Història clínica compartida de catalunya*): shared care record*; eCAP:* primary care electronic health record; HIS: hospital information system; HS (*Historial social*): social care record; SITREM (*Sistema de tractament d’Emergències*): emergency treatment;

The system maps for professional and manager sessions included behind-the-scenes system processes and pain points (Figure 4).

For Round 2, future scenarios were designed to portray a visual narrative of future interactions within 6 key themes emergent from Round 1 analysis: proximity care, empathic and quality interactions, collaborative and continuity care, personalized and proactive health prevention and promotion, active citizen participation in health, and holistic view of health. A conceptual map of the future care model vision resulting from Round 1 was another key visual used in the expert sessions.

**Figure 5.**
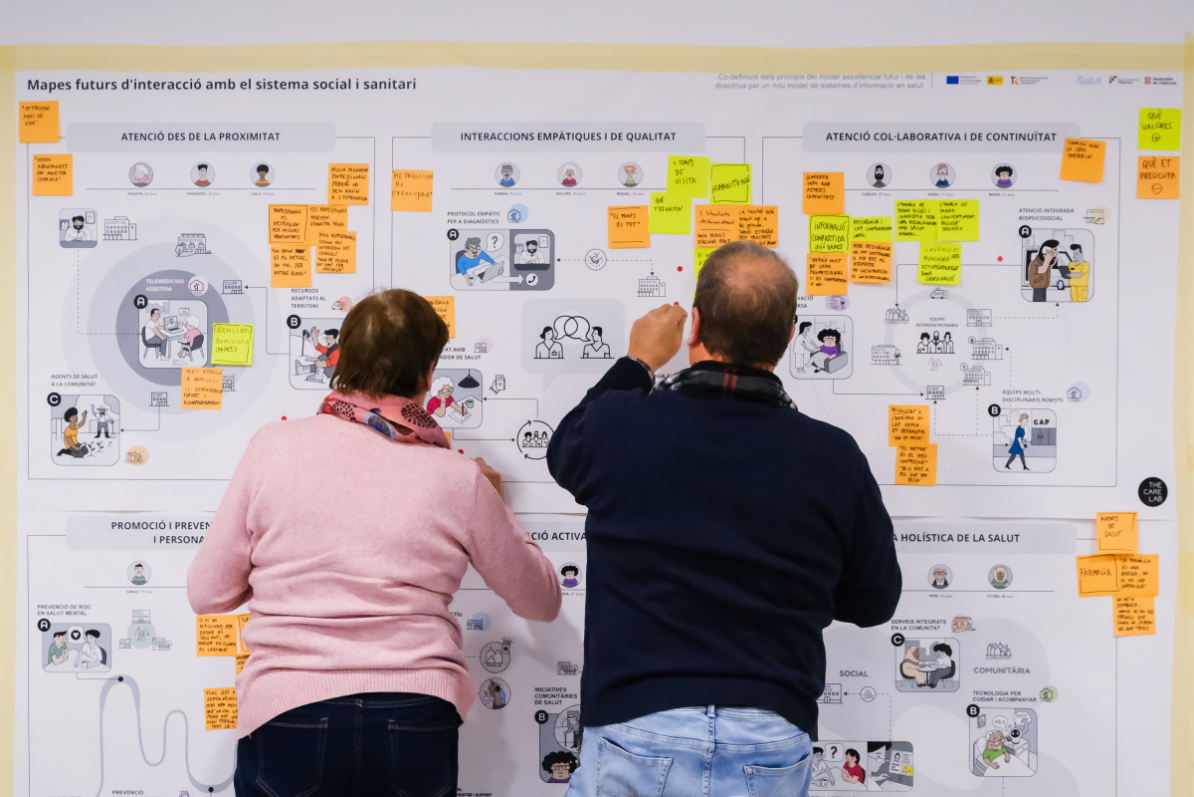
Example of a large-format poster for collective visioning with participants adding color-coded notes for item categorization.

Visual facilitation materials were carefully designed to support different communication styles and literacy levels. A facilitation toolkit was designed to enable issues to be seen at different levels of zoom or resolution, from lived experience to system overview, through technical backend, and overall delivery model. Large-format posters enabled collective visioning, while color-coded notes provided a simple yet effective method for categorizing inputs (Figure 5). Participants were given a participation kit with materials printed individually for better readability (Multimedia Appendix 1). All materials were tested for accessibility and refined based on feedback.

### Data Collection

Data collection was designed to capture both the content and context of cocreation activities through multiple complementary approaches. The facilitation team used a structured approach to data capture, with each member having specific responsibilities.

The lead facilitator maintained a neutral stance while using follow-up questions to uncover the deeper reasoning and meaning behind participants’ statements. The support facilitator observed and documented group dynamics, body language, nonverbal cues, power relations, and patterns of interaction. Dedicated notetakers captured verbatim statements, reasoning provided by participants, and contextual explanations. One notetaker focused exclusively on capturing verbatim quotes to preserve original language. Observer roles photographed and video-recorded key moments, interactions (such as voting, debating, and consensus-building), and visual outputs to document the atmosphere and participation patterns.

Multiple types of data were systematically collected from each session (1) physical artifacts including color-coded sticky notes, large-format posters, and flipcharts with participant handwriting and drawings; (2) documented narratives of participants’ lived experiences and system interactions; (3) structured observational notes on group dynamics, including dominant voices, facilitation influence, and the sequence in which ideas emerged and built upon each other; (4) photographic and video documentation; and (5) verbatim capture of participants’ oral explanations and reasoning. Table 1 summarizes the data collection methods, mapped to objectives and methods, according to project phases.

**Table 1.** Overview of methodological approaches and data collection tools by study phase.

Study phase	Timeline	Research objectives	Methods	Data collection tooling
Kick-off and Round 1 (R1) preparation	June-September 2024	Develop cocreation tools. Establish governance structure. Recruit participants. Design session protocols.	Co-design of visual tools with health experts. Tool testing and refinement.	Recruitment tracking forms. Tool design documentation. Pilot session feedback.
Round 1: current experience and future vision for the care model	October-November 2024	Understand current health care experiences. Identify barriers and facilitators. Envision desired future care model.	Journey mapping. System visualization. Experience sharing. Future visioning. Collaborative discussion.	“Pérez Family” journey tool. Current care model system maps. Color-coded sticky notes. Large-format posters. Verbatim capture. Observational notes. Photography and video. Postsession debriefs. Satisfaction questionnaires.
Synthesis R1 and Round 2 (R2) preparation	November-December 2024	Validate Round 1 findings. Define focus areas for Round 2.	Expert validation session. Collaborative synthesis.	Summary presentations. Validation discussion notes.
Round 2: future experience and design principles for future health information systems	January-February 2025	Transform needs into actionable principles. Define guidelines for health information systems. Validate and refine principles.	Scenario building. Facilitated ideation. Collaborative prioritization. Principle refinement.	Future scenario storyboards. Future care model conceptual maps. Color-coded sticky notes. Large-format posters. Verbatim capture. Observational notes. Photography and video. Postsession debriefs. Satisfaction questionnaires.
Synthesis R2 and final result	March-April 2025	Synthesize findings across rounds. Finalize design principles. Prepare dissemination materials.	Design synthesis. Thematic analysis. Principle validation.	Digital collaboration boards (Miro). Synthesis workshop outputs. Final principle documentation. Internal synthesis sessions.

Immediately following each session, the facilitation team conducted structured debriefs that captured observations on group dynamics (including identification of dominant participants, power relations, facilitation effects, and the order in which contributions emerged), surprising insights that emerged during sessions, patterns of agreement or tension, and participants’ clarifications of their contributions. These immediate debriefs served to contextualize the visual outputs while observations were still fresh, ensuring that team observations about the process of cocreation informed the interpretation of the products (visual results). The debriefs used a standardized template covering key themes that emerged, surprising insights or unexpected perspectives, group dynamics observations (including any imbalances in participation or influence), emerging patterns across different voices, and new questions arising for subsequent sessions.

Data collection was extended at least until saturation, assessed through continuous monitoring of topics. As sessions progressed, we maintained a systematic list of topics and themes being discussed across workshops. The iterative download and synthesis process allowed clustering ideas and comments around different themes for subsequent identification of both recurrent and unexplored topics. Saturation was reached when no new ideas or experiences were being captured.

### Data Analysis

We used a systematic multistage analysis process combining inductive and deductive approaches, consistent with best practices in qualitative research [52]. Analysis began immediately after each session with the structured team debriefs (steering board and specialized design practice). These direct downloads enabled key takeaways to be captured whilst fresh and vivid, allowing the methodology to be tuned between sessions to optimize the approach and outcomes and boost learnings and insights. Our front-stage and backstage teams processed detailed data in agile loops as fieldwork progressed, which avoided a build-up of data and ensured learnings were taken quickly to deepen insights.

**Figure 6.**
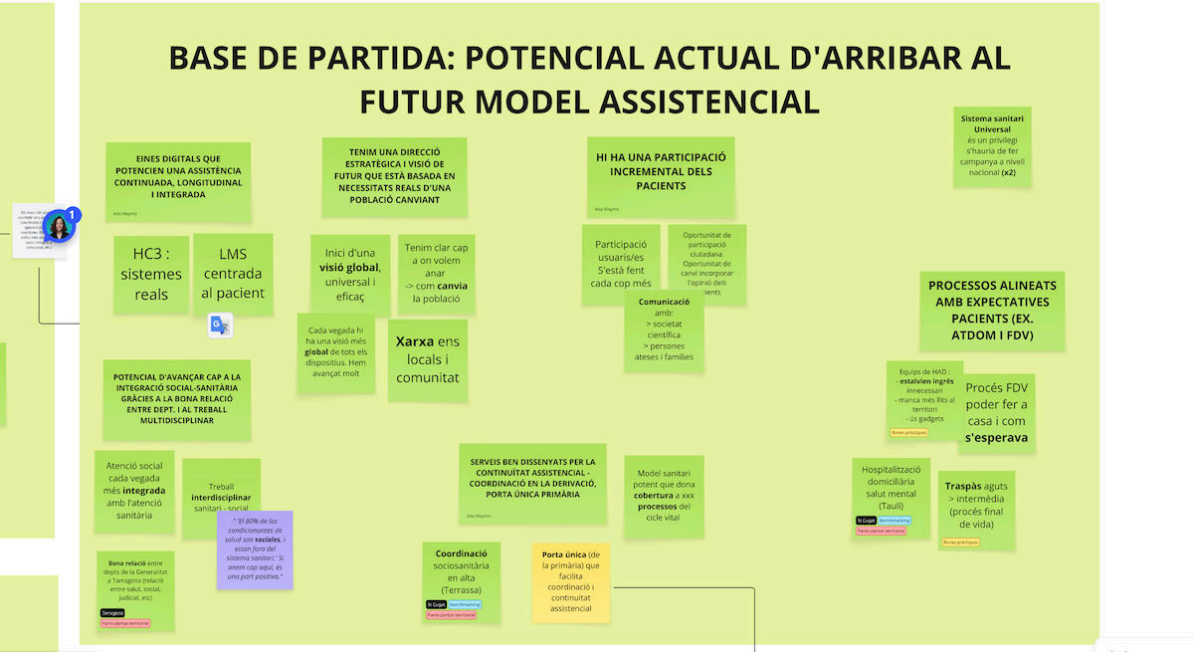
Example of digital boards (Miro software) used to analyze workshop outputs through axial coding. Contributions are mentioned in the Catalan language.

All physical materials, including colored notes, posters, and flipchart outputs, were photographed and systematically transcribed into digital collaboration boards using Miro software (Figure 6). This digitization process maintained original participant language and verbatim, preserved visual relationships between insights and ideas, and included contextual notes about discussion dynamics and nonverbal communications.

Multiple units of analysis were used. Visual outputs (colored notes and flipchart content) captured discrete participant insights and ideas. One researcher specifically documented verbatim quotes to preserve participant language. Debrief notes synthesized contextual observations about group dynamics and emergent patterns. These complementary sources enabled triangulation across data types.

Each data unit was tagged with structured metadata: geographic territory (urban, semiurban, and rural), stakeholder type (citizen, professional, manager, and expert), and workshop identifier. A systematic topic list tracked emerging themes across sessions to monitor saturation.

Analysis combined affinity mapping [53] with thematic synthesis [52]. Multiple analytical methods were integrated to ensure rigorous interpretation [54]. Initial affinity mapping clustered related insights on digital boards, grouping similar participant contributions whilst preserving their source metadata. This visual organization revealed natural patterns and relationships within the data [55]. Three researchers with complementary analytical perspectives—lived experience (citizen perspectives), operational processes (professional practice), and system architecture (organizational design)—then conducted thematic analysis. Open coding identified discrete concepts, followed by axial coding to explore relationships and create preliminary categories. Iterative team analysis sessions consolidated categories into broader themes, identifying priority challenges and opportunities.

Analytical consistency was ensured through regular synthesis sessions amongst the coding team and validation discussions with project steering board members (health information technology and clinical experts). These sessions cross-checked interpretations, identified analytical blind spots, and confirmed no significant gaps in knowledge capture.

From the Round 1 themes, we extracted foundational elements through design synthesis methods [56]. These foundations were translated into draft design principles through internal team workshops. Round 2 sessions then validated, refined, and finalized these principles through participant feedback.

### Ethical Considerations

All participants provided written informed consent, and the study received approval from the Ethics Committee of Hospital Universitari de Bellvitge (reference PR123/24). Participants were informed that their participation was voluntary and could be withdrawn at any time without consequence. Data confidentiality was maintained through anonymization procedures, and all data were stored securely with access limited to research team members. Participants provided informed consent for the recording of verbatim statements and photographs during the sessions; no images were captured for participants who rejected consent. To ensure inclusive participation, transport and professional translation services were offered when necessary. In line with the PHCD approach, we organized give-back online sessions to share the findings with participants and inform them of subsequent steps, ensuring the research delivered value to all involved.

## Results

### Overview

A total of 265 participants across the Catalan health care ecosystem participated in a 2-round PHCD process that explored current health care delivery challenges and cocreated principles for equitable digital health transformation. The findings reveal both systemic barriers and collaborative solutions emerging from systematic engagement with diverse stakeholders across Catalonia’s health care territories.

### Participant Characteristics and Territorial Distribution

Participant demographics and territorial distribution are presented in Table 2, reflecting robust representation across stakeholder groups and geographic contexts throughout the study period. The sample included citizens and informal caregivers (n=106), health care professionals (n=83), health care managers and leaders (n=50), and experts in various domains of digital health innovation (n=26). Territorial representation spanned urban centers, rural areas, and intermediate zones, ensuring diverse infrastructure and resource contexts informed the findings.

**Table 2.** Demographic characteristics of participants.

Participant characteristics		Values, n (%)
Patients and caregivers (n=106)		
Gender		
Men		39 (36.8)
Women		67 (63.2)
Age (years; missing=9; n=97)		
18‐35		2 (2.1)
36‐50		16 (16.5)
51‐65		38 (39.2)
>65		41 (42.3)
Profile (n=106)		
Patient		68 (64.2)
Caregiver		38 (35.8)
Territorial context		
Urban		20 (18.9)
Semiurban		49 (46.2)
Rural		37 (34.9)
Health and social professionals, experts, and leaders (n=159)		
Gender		
Male		57 (35.8)
Female		102 (64.2)
Age (years; n=159)		
18‐35		7 (4.4)
36‐50		100 (62.9)
51‐65		52 (32.7)
>65		0 (0.0)
Profile (n=159)		
Family physician		4 (2.5)
Hospital and intermediate care physician		16 (10.1)
Primary care nurse		11 (6.9)
Hospital and intermediate care nurse		9 (5.7)
Psychology		4 (2.5)
Social worker		7 (4.4)
Health administration		7 (4.4)
New roles (community well-being, physiotherapist, and nutritionist)		7 (4.4)
Others (emergency service, pharmacy and care homes)		13 (8.2)
Health care managers and leaders		50 (31.4)
Experts in digital health innovation		26 (16.4)
Territorial context		
Urban		91 (57.2)
Semiurban		48 (30.2)
Rural		20 (12.6)

**Figure 7.**
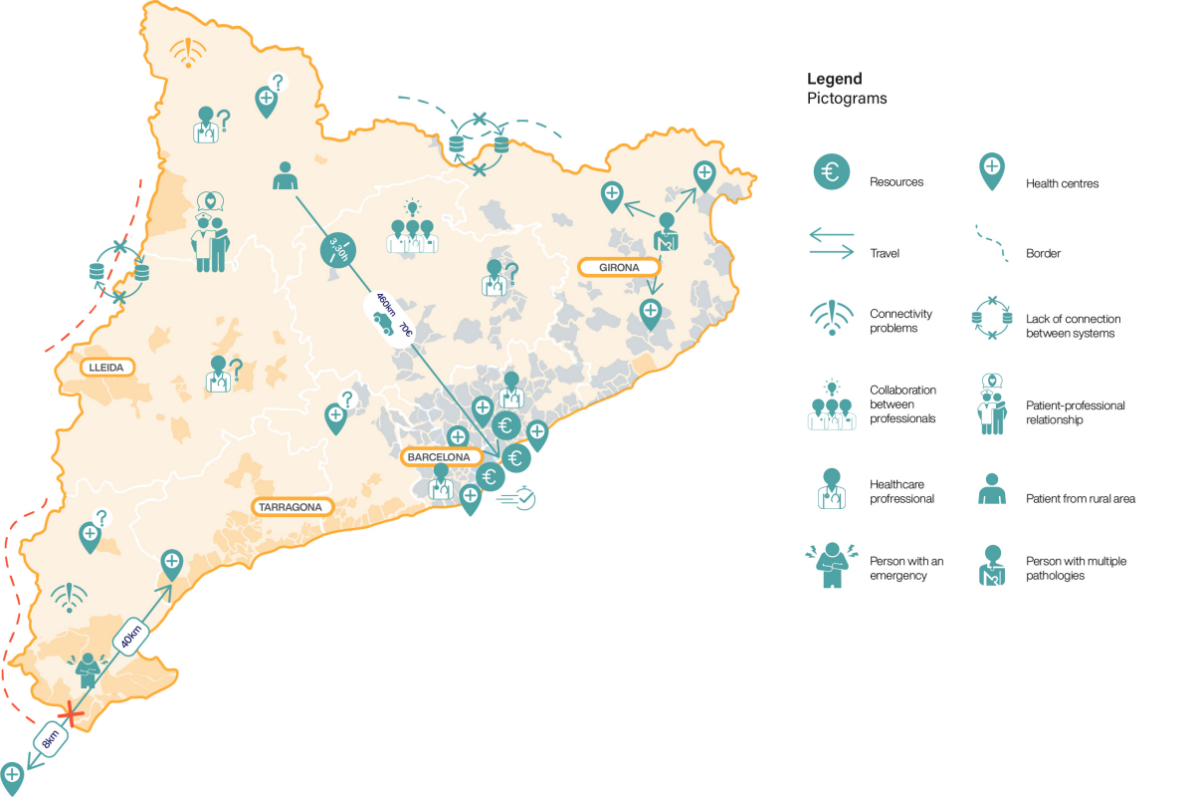
Summary of barriers to digital health equity identified during Round 1, contextualized per geographic area.

Through journey mapping and storytelling activities, Round 1 participants articulated 13 priority barriers to digital health equity that span technological, organizational, and sociocultural dimensions. These barriers emerged consistently across urban, semiurban, and rural contexts, though with varying emphases reflecting territorial specificities (see Figure 7 for territorial variations).

While these barriers emerged across all territories, their manifestation and intensity varied by geographic context. Rural areas emphasized connectivity and resource access challenges; urban contexts highlighted diversity and complexity issues; border regions contributed unique perspectives on administrative complexity and cross-jurisdictional care. Table 3 presents the 13 priority barriers with illustrative participant quotes demonstrating lived experiences across these diverse contexts. Detailed descriptions of all 13 barriers with extended context and additional participant quotes are available in Multimedia Appendix 2.

**Table 3.** Barriers to digital health equity identified in round 1, with illustrative participant quotes.

Barrier^a^	Description	Participant quote^b^
Fragmented health and social care integration	Services function in a fragmented manner due to hyperspecialization, generating multiple referrals that break care continuity. Primary care, under pressure, cannot assume its connecting role.	“Everything to do with the social system is a black box. I don’t know what happens there. And we should know! 80% of health determinants are social*.*” [Health care manager]
Insufficient emotional well-being support	Growing need for emotional support with a preventive, holistic, and community approach. Current system designed primarily for emergencies and physical pathologies.	“Community networks and neighbors are great prescribers of health and could be protective and informative elements.” [Health care manager]
New citizen expectations are creating system pressure	Citizens have growing expectations about immediacy, flexibility, and personalization influenced by digital society, generating pressure on a system lacking resources to respond.	“We need to move towards a one-stop-shop system. It will make life easier for both the user and us.” [Health care manager]
Lack of health education and prevention	Many people lack knowledge about self-care, prevention, and appropriate use of health services, leading to system misuse (eg, A&E saturation for nonurgent cases).	“Health literacy is fundamental. If people don’t understand their health, they can’t manage it properly.” [Health care professional]
Social, language, and digital barriers	Digital gaps, medical terminology, and language barriers prevent many from accessing and navigating the system, especially older adults and migrant populations. Overall, 48% of the foreign population is at risk of poverty or social exclusion.	“Many times I don’t understand the doctors’ calls. If they give me a biopsy result I’m none the wiser, or I have to say, please, tell me in words I can understand. I prefer to go to the place rather than receive a call*.*” [Service user]
Insufficient resources for aging and complexity	Progressive aging brings increased care needs for people with frailty and complexity. The current system lacks sufficient human and material resources for continuous, specialized, often home-based care.	“Resources should be moved towards the patient, rather than moving the patient towards resources. For example, prioritizing home care over residential facilities.” [Health care manager]
Waiting lists with human consequences	Long waiting lists carry health risks and a strong emotional impact. Overall, 64% of patients wait >5 days to access a family physician; 150 days average for surgical intervention. Many are forced to turn to private health care, generating access inequalities.	“I’m an electrician, I’m self-employed, and they were giving me a physio appointment in 8 months. I can’t wait, what will I eat? If you don’t have private healthcare, you’re done for.” [Service user]
Resource planning based on quantitative criteria	Planning and resource allocation based on quantitative criteria (number of appointments, procedures) rather than health outcome indicators, leading to inadequate incentives.	“As a professional, you make the diagnosis, but when it comes to implementing truly, it’s not possible because a specialist is missing. Something behind there should be a map of specialists that lets us zoom in and see, for example, the difference between supply and demand in areas like allergies and dermatology.” [Health care professional]
Primary care unable to assume connecting role	Primary care should coordinate and ensure continuity, but pressure, lack of resources, and system fragmentation prevent it from exercising this role, generating breaks in care continuity.	“Primary Care must be the real entry and staying point for care. It shouldn’t be a referral center. We need to do good work and strengthen Primary Care more.” [Health care professional]
Inadequate protocols for chronicity and mental health	System excels at emergencies but lacks reinforcement in continuous preventive care and adequate support for daily management of chronic diseases. Overall, 37.8% of the adult population has chronic illness; 9.84% increase in chronic mental health patients since 2017.	“Mental health is left to God, more resources should be allocated to it. [...] Pure mental pathology is ‘the great forgotten.’ It works well in critical moments and acute hospitalization, but it doesn’t have enough resources for chronic, continuous care within the community.” [Service user and Health care professional]
High professional turnover	High turnover due to work culture changes and stressful conditions significantly affects care quality and continuity. Rotation prevents creating trust bonds essential for effective care, particularly impacting older patients and those with mental health needs.	“My psychologist has changed every other time. Each time you go you have to expose yourself again and explain what’s happening to you.” — Service user"I’ve even thought about leaving medicine and changing jobs. I work loads of hours, and you have no help from anywhere. I’m taking diazepam for anxiety.” [Health care professional]
Administrative burden compromising care	Health care professionals dedicate significant time to administrative and bureaucratic tasks, reducing time for direct patient care, compromising care quality, and contributing to staff burnout.	”There should be an automatic process that allows you to spend more time with the patient. Here some technology like AI or a different way of entering information could play an important role.” [Health care manager]
Multiple noninteroperable information systems	Multiple noninteroperable systems hinder clinical information access and sharing between professionals from different care settings, generating inefficiencies, duplications, risk of errors, and coordination difficulties.	”Information should be accessible to all professionals because the information belongs to the patient, not to the professionals.” [Health care professional]

a Barriers synthesized from 189 participants across 19 round 1 sessions (16 territorial cocreation sessions +3 expert interviews).

b Quotes translated from Catalan/Spanish.

### Cocreated Design Principles for Equitable Health Information Systems

**Figure 8.**
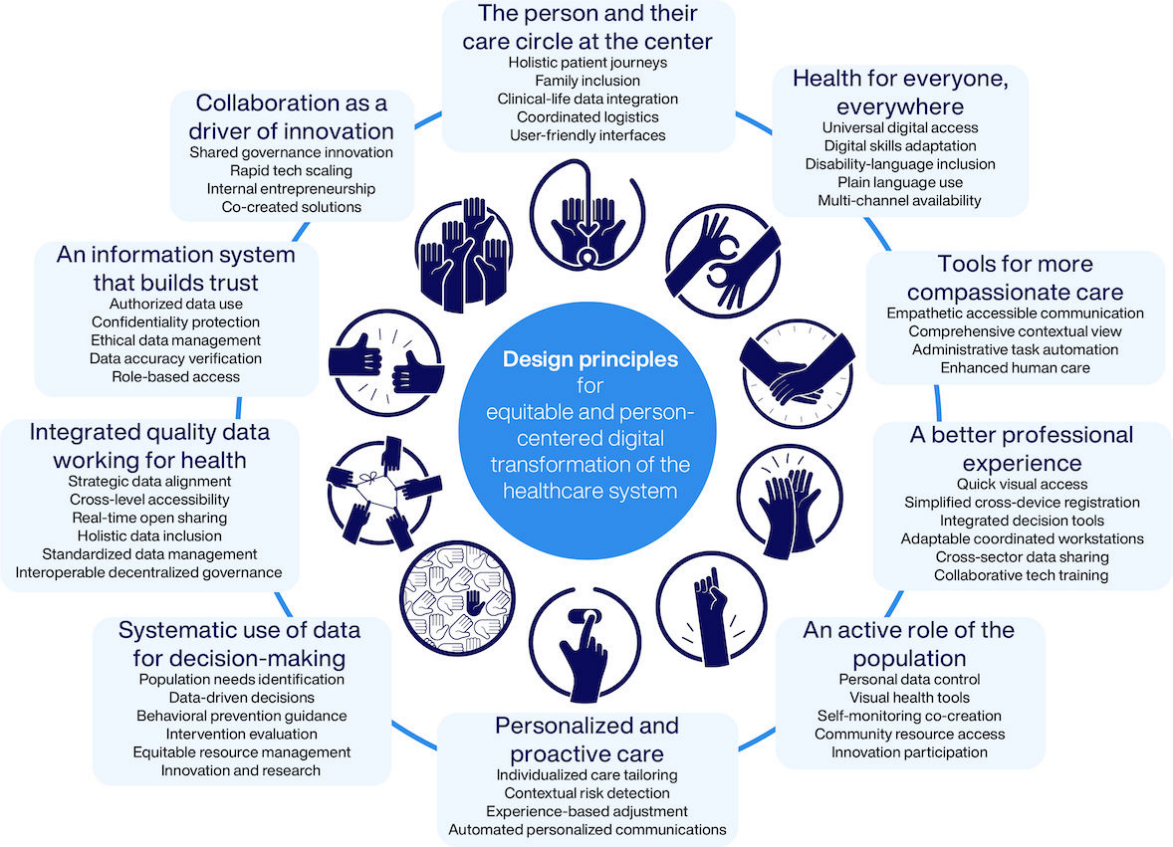
Cocreated fundamental principles for equitable digital health information systems.

Round 2 involved 8 territorial cocreation sessions, one validation session with Catalan Health Service experts, and 3 topic-specific sessions. Building on barriers identified in Round 1, participants cocreated 10 fundamental principles for equitable health information systems that address technological, organizational, and social dimensions of digital health equity (Figure 8).

### Territorial Context and Principle Development

Territorial analysis revealed how geographic and infrastructure contexts shaped both barrier experiences and the development of information system design principles. Rural areas emphasized connectivity and resource access issues, leading to a stronger emphasis on principles addressing “Health for Everyone, Everywhere” and “Collaboration as a Driver of Innovation.” Urban contexts highlighted diversity and complexity challenges, informing principles focused on cultural responsiveness and care coordination. Border regions contributed unique perspectives on administrative complexity and cross-jurisdictional care access, strengthening principles addressing system integration and user navigation support. These territorial differences informed the development of principles that explicitly address contextual variation rather than assuming standardized solutions.

### Stakeholder Perspective Alignment and Divergence

While remarkable alignment emerged across stakeholder groups on fundamental principles, meaningful divergences in emphasis provided valuable insights for implementation. Health care professionals prioritized workflow integration and administrative burden reduction more strongly than other groups, directly informing the principle of “A Better Professional Experience.” Patients and caregivers emphasized ease of use and personal control over health information, strengthening principles focused on user empowerment and data governance. Health care managers and leaders focused on sustainability and implementation feasibility, contributing to principles addressing systematic data use and collaborative innovation. Experts in digital health innovation highlighted technical interoperability and innovation potential, enriching principles related to data integration and system connectivity.

Although rare, certain divergences revealed conflicting views on the same topic. For instance, regarding mental health data sharing, parents expressed concerns about schools accessing their children’s information, while professionals emphasized the need for cross-team coordination to optimize care. Such tensions were addressed by exploring underlying reasoning during facilitation and using divergences generatively during synthesis. Rather than forcing consensus, conflicting perspectives directly informed principles that could respond equitably to multiple stakeholder needs, such as Principle 9 on data governance, which emphasizes individual control over context-specific information sharing.

### Implementation Considerations

Participants not only cocreated principles but also provided detailed insights into implementation considerations. They emphasized the need for phased implementation approaches that build confidence and capability gradually, strong change management and training programs for both citizens and health and social care professionals, and maintaining human connection even as systems become more automated. The cocreation process itself was identified as a valuable model for ongoing stakeholder engagement throughout implementation, with participants requesting continued involvement in system development and evaluation.

### Participation Experience and Engagement

Participants demonstrated high levels of engagement throughout the cocreation process. Postsession evaluation surveys indicated strong satisfaction with the participatory experience, with participants reporting that they felt heard, respected, and valued in the process. The visual facilitation tools, particularly the “Pérez Family” journey mapping and storytelling, proved effective in enabling participants with varying communication styles and literacy levels to contribute meaningfully to discussions while providing a common visual framework upon which diverse experiences, ideas, and perspectives could be consolidated to create a shared mental model. The diversity of profiles facilitated rich exchanges between different perspectives, with participants frequently noting the value of hearing experiences from other stakeholder groups.

## Discussion

### Principal Findings

In this study, PHCD allowed us to generate a deep understanding of lived experiences and challenges faced by diverse stakeholders navigating the health care ecosystem in Catalonia, while simultaneously cocreating principles for guiding equitable digital transformation. Through engaging citizens, caregivers, professionals, managers, and different expert profiles in collective reflection and design, the process not only uncovered structural and experiential barriers but also translated these insights into actionable principles for future health information systems. Finally, the implemented and documented large-scale, iterative co-design approach provides a replicable methodology that other health systems can adapt to embed equitable care supported by health information technology as a central value in their transformation processes.

Our participatory approach yielded ten design principles that address critical gaps in current digital health development. These principles emerge from a comprehensive understanding of real-world challenges faced by patients, caregivers, health care professionals, and system leaders across diverse territorial contexts. The cocreation process revealed that digital health equity requires attention to multiple interconnected dimensions: technological accessibility, organizational readiness, professional workflow integration, and citizen empowerment in data governance.

The barriers identified (eg, digital literacy gaps, fragmented information systems, and insufficient consideration of vulnerable populations) align with the Digital Health Care Equity Framework in the United States [27], which similarly emphasizes addressing digital determinants of health through participatory design and community engagement. Our findings extend this work by showcasing how large-scale participatory processes can systematically translate these equity considerations into actionable design principles for driving digital transformations in the health care system.

The 10 principles address long-standing challenges in health IT development, particularly the gap between technological capabilities and real-world usability. Recent research continues to document usability problems, with over one-third of medication safety events in pediatric hospitals related to EHR usability issues[57].

The principle of “The Person and their Circle at the Center” reflects growing recognition that meaningful stakeholder involvement throughout design is essential [58]. This aligns with evidence from an umbrella review identifying participatory design and community collaboration as crucial for creating digital health tools serving diverse user needs [59]. Our study extends this evidence by scaling the community collaboration across an entire regional health system and applying it to define core design principles.

The emphasis on data control and citizen agency in the principle “An Information System that Builds Trust” resonates with recent research on patient preferences for health information exchange design, revealing complex attitudes toward health information control and supporting our finding that genuine patient engagement is essential for addressing privacy and trust concerns [60].

### Knowledge Contributions Beyond Design Artifacts

While producing 10 specific design principles, our research contributes to understanding digital health equity beyond these outputs through three key areas.

First, our findings illustrate how digital health equity barriers operate across multiple interconnected levels in practice. Previous frameworks have conceptualized equity barriers at individual, interpersonal, community, and societal levels [26,28]; implementation guidance has often remained siloed [22,29]. Our participatory process documented how stakeholders experience these barriers as deeply interconnected: technically accessible interfaces are undermined when organizational training support is missing; well-designed systems fail when procurement policies exclude user input; individual digital literacy interventions have limited impact when infrastructure gaps persist. In this regard, our findings suggest that equity-centered transformation may require coordinated technical, organizational, and systemic change because barriers appear to operate synergistically across these dimensions.

Second, our study documents how participatory design processes can serve as more than requirements-gathering exercises. Traditional health IT development treats stakeholder engagement as a requirements-gathering phase that feeds into expert-driven design [5,6]. Our experience indicates that participatory processes may generate equity-relevant outcomes through several mechanisms: surfacing barriers invisible to system designers [10], legitimizing experiential knowledge alongside technical expertise [18], and building stakeholder investment in transformation [59].

Finally, our research contributes to growing evidence highlighting the challenge of retrofitting equity considerations, which instead need to be embedded from the outset. The barriers identified appeared to stem not from isolated technical shortcomings but from design processes that systematically excluded diverse users [61]. Therefore, increasing equity may require restructuring design processes themselves, shifting from technology-driven implementation, where solutions are predetermined, to genuinely participatory cocreation, where diverse voices shape problem definition, solution ideation, and implementation priorities [14,15,21]. This concept aligns with that of other authors claiming that design processes themselves can either reproduce or mitigate existing health inequities [1,2,10].

### Limitations and Transferability

While our study provides valuable insights into participatory design for digital health equity, the external validity of our principles and conclusions may be influenced by intrinsic features of the study design.

The geographic focus on a single area under the same health care system (ie, Catalonia) may have led to some insights not applicable to other countries and thus limiting transferability. However, we consider most of our findings applicable and valuable beyond regional boundaries through several dimensions. First, many of the identified barriers represent universal systemic challenges (workforce shortages, resource constraints, service fragmentation, and navigation complexity) documented across diverse health care contexts. Second, the methodological framework itself is transferable due to the inclusion of territorial segmentation (rural vs urban contexts) and multilevel stakeholder engagement (citizens to policymakers and health care to social care), which are categories relevant to most health systems. Third, specific findings regarding data duplication between public and private providers are particularly relevant and common in mixed-model health care systems, which are increasingly prevalent globally. Finally, the governance of the Catalan health system is regionally commissioned, like other environments, such as England’s Integrated Care Boards, enhancing organizational-level transferability. Future research should examine how these principles perform across different health care contexts, particularly in low-resource settings where digital equity challenges may be more pronounced.

Our study captured stakeholder perspectives at a specific point during rapid digital transformation. The long-term sustainability and relevance of identified principles will require ongoing evaluation as digital health technologies and user needs evolve. However, we attempted to ensure future-proofing through grounding principles in fundamental human-centered design values, focusing on equity considerations that persist regardless of specific technologies, and validating principles through diverse stakeholder perspectives representing enduring health care needs.

Another feature that may influence generalizability is the participant profile involved in the sessions. Despite our effort in recruiting a diverse profile regarding roles, geographic location, and demographic characteristics, the voluntary nature of participation may have introduced selection bias towards individuals with a stronger interest in digital health transformation. This was particularly relevant for migrant and underserved communities, which were underrepresented. These populations often face the most significant barriers to digital health access. The exclusion of these voices represents a significant limitation, as these communities are frequently most affected by digital health inequities.

Future research could explore mechanisms for engaging harder-to-reach populations, with particular attention to developing culturally appropriate recruitment strategies for migrant communities and other underserved populations.

### Implications for Practice and Policy

Our findings have important implications for health care organizations, technology vendors, and policymakers. For health care organizations, our study highlights the value and feasibility of investing in meaningful participatory processes before, during, and after health information system implementations. The principles provide a framework for evaluating existing systems and guiding future technology decisions with explicit attention to equity considerations.

A critical insight is the importance of innovative partnership approaches when navigating system complexity to drive large-scale engagement and co-design processes. In our experience, successful system-level transformation requires sophisticated collaboration models bringing together diverse expertise and perspectives. The partnership between public health authorities, academic institutions, and specialized design practitioners created a unique configuration enabling navigation of complex health care system dynamics whilst maintaining methodological rigor and stakeholder trust. This proved essential for accessing diverse participant networks, managing territorial complexities, and ensuring that cocreated principles would be both evidence-based and implementable within existing structures.

For technology vendors, our research highlights the importance of embedding human-centered design processes throughout product development, not merely as compliance exercises. Persistent usability problems documented in EHR research suggest current vendor approaches to stakeholder engagement are insufficient [52]. Our study provides evidence that more comprehensive participatory approaches can yield insights leading to more effective, equitable, and sustainable system designs.

For policymakers, our findings support the need for regulatory frameworks that incentivize or require meaningful stakeholder engagement in health technology development. Our study demonstrates the feasibility and value of comprehensive participatory approaches that could inform future policy development. Additionally, policymakers should consider how to support and incentivize the development of collaborative partnerships and co-design capabilities within health care systems, recognizing that these represent critical infrastructure for equitable digital transformation.

### Future Directions

The 10 principles provide a foundation for future research examining their implementation and effectiveness in practice. Critical areas include extending these principles to social and community care settings, examining how they apply to artificial intelligence–enabled health technologies [62], and exploring how they can inform the development of community health information systems. Implementation research examining how organizations can operationalize these principles, including necessary capabilities, resource requirements, and implementation strategies, would provide valuable guidance for translating insights into improved health information systems.

### Conclusions

This study showcases how PHCD, conducted at a large scale and grounded in rigorous methodology while engaging all relevant stakeholders, can serve as a powerful tool to identify equity barriers in health information systems and inform relevant and specific principles driving digital transformation of health care systems. The 10 principles emerging from this study provide a comprehensive framework for developing person-centered health information systems that are not only technically robust but also genuinely responsive to user needs, culturally appropriate, and accessible to all populations.

This study makes three distinctive contributions that differentiate it from existing research. First, it is the first successful application of PHCD at a regional health system scale, substantially exceeding the typical scale of participatory design studies in health care. Second, it provides empirical evidence of how equity barriers operate synergistically across technical, organizational, and systemic levels in practice, challenging the siloed implementation approaches suggested by previous conceptual frameworks. Third, it exemplifies how participatory processes can serve as transformative mechanisms for embedding equity from the outset, rather than the requirements-gathering exercises that characterize traditional health IT development. These contributions provide both a methodological blueprint for scaling participatory approaches and evidence that restructuring design processes themselves is essential for achieving digital health equity.

## Supplementary material

10.2196/84129 Multimedia Appendix 1 Participant selection and recruitment strategy, co-design materials, and participation surveys.

10.2196/84129 Multimedia Appendix 2 Round 1 outcomes (extended version).

## Abbreviations

EHR: electronic health record
PHCD: participatory human-centered design
SRQR: Standards for Reporting Qualitative Research
WHO: World Health Organization
