# Supplementary material for: Cocreating Principles for Digital Health Equity: Cross-Sectional, Qualitative Study for Participatory Human-Centered Design in Catalonia
Source: J Med Internet Res. 2026 Jan 6;28:e84129. doi: 10.2196/84129 (PMC12774400; doi:10.2196/84129)
Supplement: Multimedia Appendix 1 [file jmir-v28-e84129-s001.pdf]

Co-creating principles for digital health equity: A cross-sectional, qualitative study for participatory human-centred design in Catalonia

Supplementary material

- Participant Selection and Recruitment
- Familia Perez, full version poster for Round 1
- Future scenarios for co-creation Round 2
- Collective vision for the evolution to the future care model
- Participation survey – citizens & caregivers
- Participation survey – professionals

**All translated to English from the original version*

Participant Selection and Recruitment

Participant Selection and Recruitment

1. Overview and Rationale

Participant selection and recruitment followed a purposive sampling strategy aimed at ensuring broad, equitable, and meaningful representation across all relevant actors of the Catalan health and social care system. The purpose was to guarantee that the co-creation process would reflect diverse lived experiences, operational realities, and strategic perspectives, enabling the development of a care-process model and digital health design principles that respond to the real needs of all stakeholders.

All participants had an established connection to the Catalan Health System—either as service users, informal caregivers, frontline professionals, organisational managers, or experts in strategy, innovation, and digital health.

Recruitment adhered to the four core criteria of the Framework for Citizen Participation in Health of the Catalan Department of Health:

(1) adequate methodological definition,

(2) scientific and ethical rigor,

(3) protection of vulnerable and minority populations, and

(4) sustainability of the public health system.

2. Stakeholder Groups Included

The study targeted four main stakeholder groups, representing micro, meso, and macro levels of the system. These groups and their selection criteria are described below.

2.1. Citizens and Informal Caregivers (Micro Level)

Participants included adults with lived experience navigating the health and social care system. They represented diverse profiles such as:

- Patients with acute, chronic, or complex conditions
- Informal caregivers (family members, neighbours, community carers)
- Expert patients, peer-support leaders, or representatives of patient associations

Selection criteria included:

- Representation across urban, semi-urban, and rural territories
- Diversity in age, gender, socioeconomic status, disability status, and migration background
- Capacity to share lived experiences in workshops or group sessions
- Participants contributed insights into access, continuity, care coordination, communication, administrative burden, and challenges in digital engagement.

2.2. Health and Social Care Professionals (Micro Level)

Professionals were selected to represent a wide range of roles and care settings. Profiles included:

- Primary care physicians and nurses
- Hospital and intermediate care physicians and nurses
- Social workers, psychologists, pharmacists
- Administrative personnel
- Emergency care professionals
- Professionals in new or emerging roles (physiotherapists, nutritionists, community wellbeing agents)

Selection criteria included:

- Representation across care levels: primary, hospital, intermediate, emergency, social care
- Employment in public, private, or non-profit organisations
- Experience in daily care delivery and familiarity with coordination challenges
- Ability to participate in workshops, group interviews, or transversal sessions
- Professionals contributed frontline perspectives on workflow, coordination barriers, information system fragmentation, and professional burden.

2.3. Territorial Managers, Sector Directors, & Provider-level Leadership (Meso Level)

This group included individuals holding managerial, organisational, or strategic responsibilities across Catalonia.

Profiles included:

- Territorial health managers
- Sector directors
- Directors and managers of healthcare and social care provider organisations
- Organisational leaders involved in digital transformation or integrated care planning

Selection criteria included:

- Representation across all ten health regions
- Involvement in strategic planning, resource allocation, or operational management
- Capacity to identify structural constraints and opportunities in the existing model
- Managers contributed insights into operational feasibility, sustainability considerations, and system-wide barriers and enablers.

2.4. Experts in Strategy, Innovation, and Digital Health (Macro Level)

A fourth group included specialised experts able to provide broader system and technical perspectives.

Profiles included:

- Experts in digital health strategy
- Specialists in interoperability and information systems
- Innovation consultants
- Representatives of quality, evaluation, and digital health agencies

These experts refined the analysis, validated findings, and assessed feasibility of proposed design principles.

3. Recruitment Process

3.1. Role of Territorial Health Coordinators

Recruitment was operationalised through health coordinators of the ten Catalan health regions, who act as managerial leaders responsible for translating Catalan Health Service policies into regional practice.

Their responsibilities included:

- Identifying potential participants using explicit diversity criteria
- Ensuring representation of the four stakeholder groups
- Prioritising inclusion of vulnerable and under-represented populations (older adults, immigrants, people with disabilities, low socioeconomic status)
- Coordinating logistics and communication with selected participants
- Participating in training and alignment meetings with the research team

All coordinators attended an initial kick-off meeting in which the research team presented the study aims, methodology, eligibility criteria, and recruitment procedures.

Weekly monitoring meetings with the project manager ensured consistency and recruitment progress across regions.

3.2. Purposive Sampling Strategy

The sampling strategy was designed to maximise diversity across:

- Territorial context: urban, semi-urban, rural
- Organisational types: public, private, and third sector providers
- Care levels: primary, hospital, intermediate, emergency, and social care
- Sociodemographic diversity: age, gender, migration status, socioeconomic status, disability
- Professional roles: clinical, social, administrative, management, and technical experts

Special emphasis was placed on intersectional diversity, recognising that healthcare barriers emerge through the interaction of multiple factors (territory, gender, age, socioeconomic status, etc.).

4. Territorial Selection

To ensure representation of the Catalan territory, sessions were conducted across a balanced distribution of regions. Locations were selected collaboratively with territorial coordinators to capture contextual variability in:

- infrastructure and digital access
- population density
- resource availability
- care organisation and intersectoral relations

Urban, semi-urban, and rural settings were intentionally included across both rounds of data collection.

Familia Pérez, full version poster for Round 1

Finançat per la Unió Europea NextGenerationEU
Plan de Recuperación, Transformación y Resiliencia
/Salut
Next Generation Catalunya
Generalitat de Catalunya

The life of the Pérez family and their relationship with the social and health system throughout life

CONTACT POINTS / SERVICES

La Meva Salut	Primary Care	Pharmacy	Day Hospital	Specialized outpatient care	Intermediate care	Social + Home care Services
Telephone Attention	Home Care	Emergency Services	Therapeutic Care	Hospitalization	Day Center	

Aquí s'ha de definir què és PADES, CUAP, ASSIR... Potser hem de fer les traduccions primer: Home Care Program, emergency primary healthcare centre ,Attention to Women's Sexual and Reproductive Health etc... i algunes no abreviar-les. Si les abreviem, definir-les al peu de figura. Aquí necessitem l'Alèxia per l'editable.

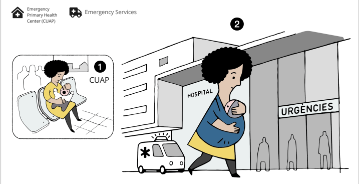

Rosa and Nil, 30, daughter / Barcelona
Single mother, just gave birth. Both the follow-up and the delivery were at the Sant Joan de Déu hospital. Today she had to go to the emergency room because the baby has a fever of 40 and it won't go down. After going to the emergency room at CUAP, she decides to go to the emergency room at the Sant Pau hospital because the fever remains high.

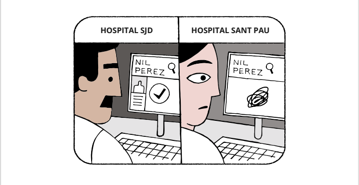

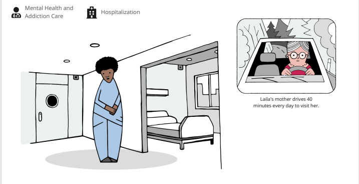

Laila, 19, niece / Sort
Two weeks ago, she was admitted to the adult unit of Mental Health in Tremp due to a crisis from the eating disorder she has suffered from since childhood. There were no places available at the day hospital in her town.

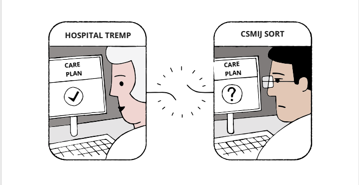

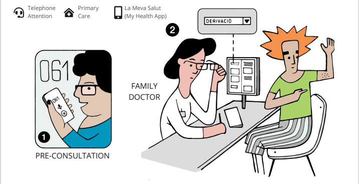

Miquel, 23, son / Mataró
His mother called 061 to inquire about a possible allergy. The family doctor refers him to the allergist, which will take about six months. Additionally, the nurse from the CAP shares information with him to prevent obesity, as he has gained a bit too much weight.

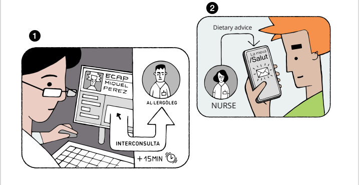

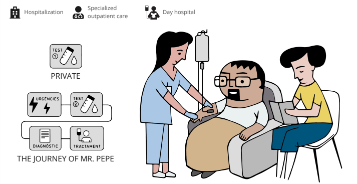

Pepe, 55, friend / Olot
After three months undergoing tests at a private clinic for chest pain, he is hospitalized for bronchitis at the Olot Hospital, where some of the tests he had already undergone are repeated, leading to a diagnosis of lung cancer. He will have to go weekly to Trueta in Girona for treatment. His wife will accompany him.

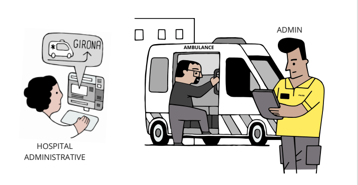

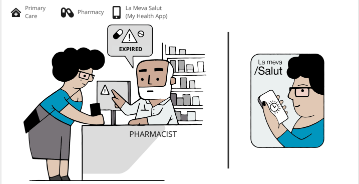

Maria, 57 / Mataró
She has a check-up with the family doctor because she has diabetes and will have the results of the tests in a week on La Meva Salut. After the visit, she goes to the pharmacy to pick up her medication and takes the opportunity to collect her mother's, but it is expired.

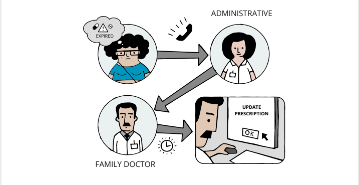

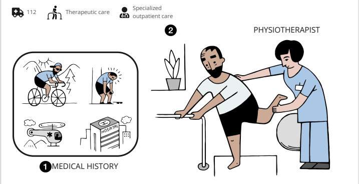

Joan, brother, 62 / Arenys de Mar
While on vacation in Vielha, he suffered a stroke and was airlifted to Vall d'Hebron. He has just started rehabilitation in Mataró and has video call check-ups with the specialist. Joan will need to adjust his diet so it does not interfere with the new medication.

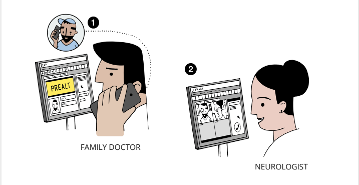

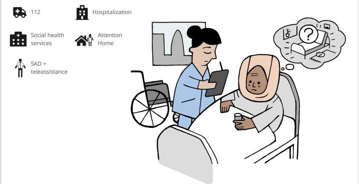

Fàtima, neighbor, 80 / Lives alone in Mataró
A month ago, she fell and broke her hip while going down the stairs. Maria called 112 and she was taken to the hospital for emergency surgery. After being discharged, she stayed at the social health center for a few days and now has the AtDom team, which visits her at home to manage her pain, and the SAD, which supports her with daily tasks while she recovers.

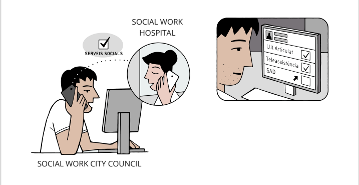

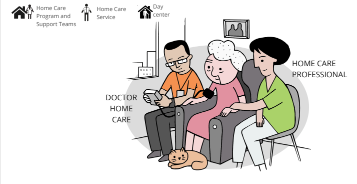

Paquita, mother, 87 / Lives with Maria in Mataró
She has dementia, arthritis, and heart failure for the past eight years. Since her daughter works, a caregiver helps her with household tasks and she goes to the Day Center every week. The last decompensation left her very fragile and Home Care Program has been activated to provide her comfort.

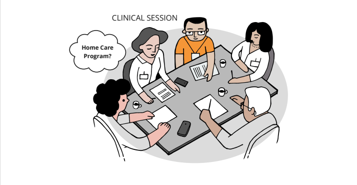

Future scenarios for co-creation Round 2

PROXIMITY CARE

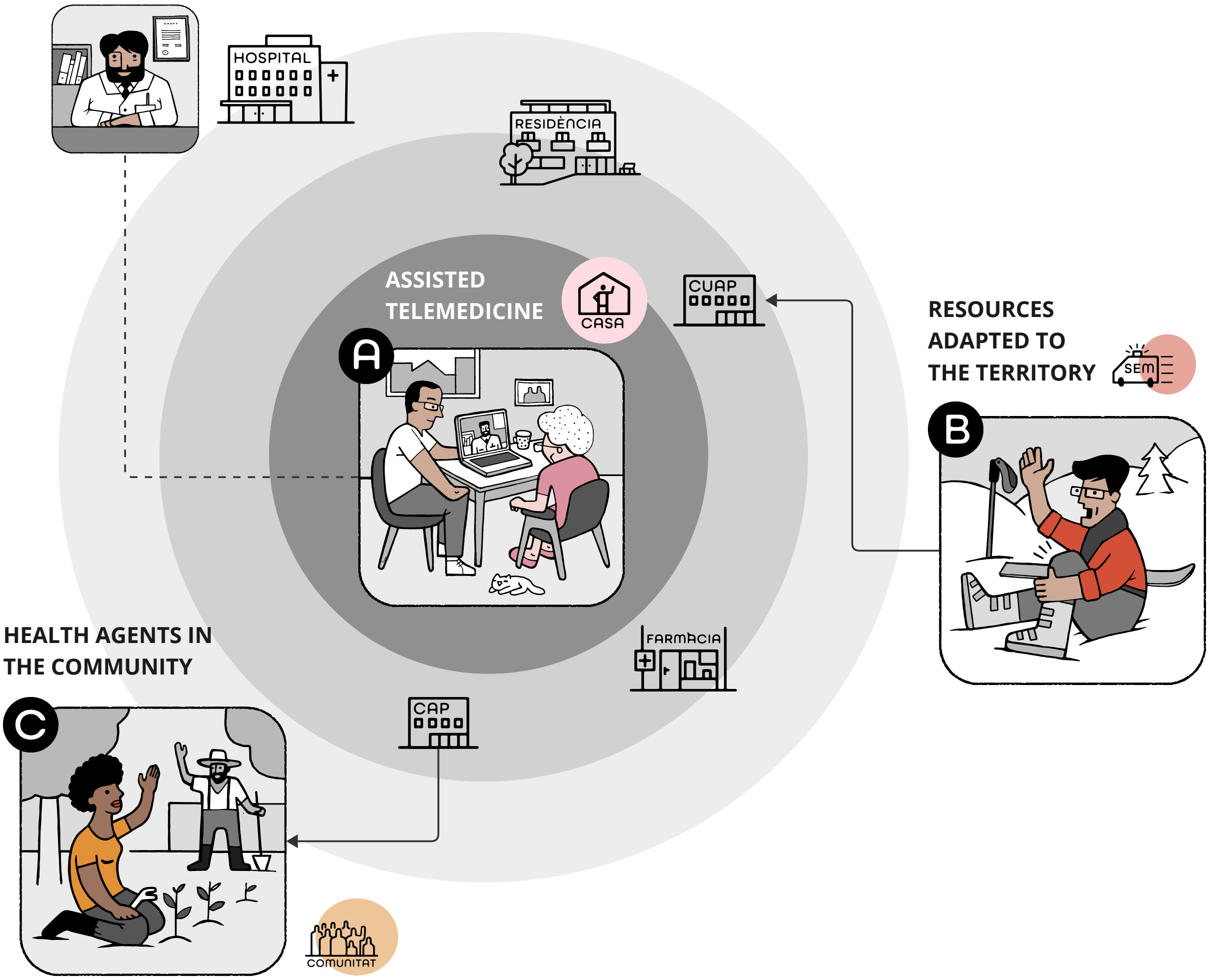

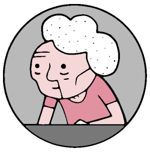

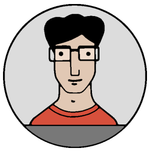

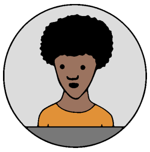

PAQUITA, 93 years old

Mataró

FRANCESC, 38 years old

Cerdanya

LAILA, 29 years old

Tremp

A future scenario in which attention from proximity is understood through two avenues: promoting the nearby environment, with primary and community care as the central axis of assistance, and distributing resources dynamically across the territory thanks to new technologies, all while ensuring equitable and accessible care, where services reach where people are located.

EMPATHIC AND QUALITY INTERACTIONS

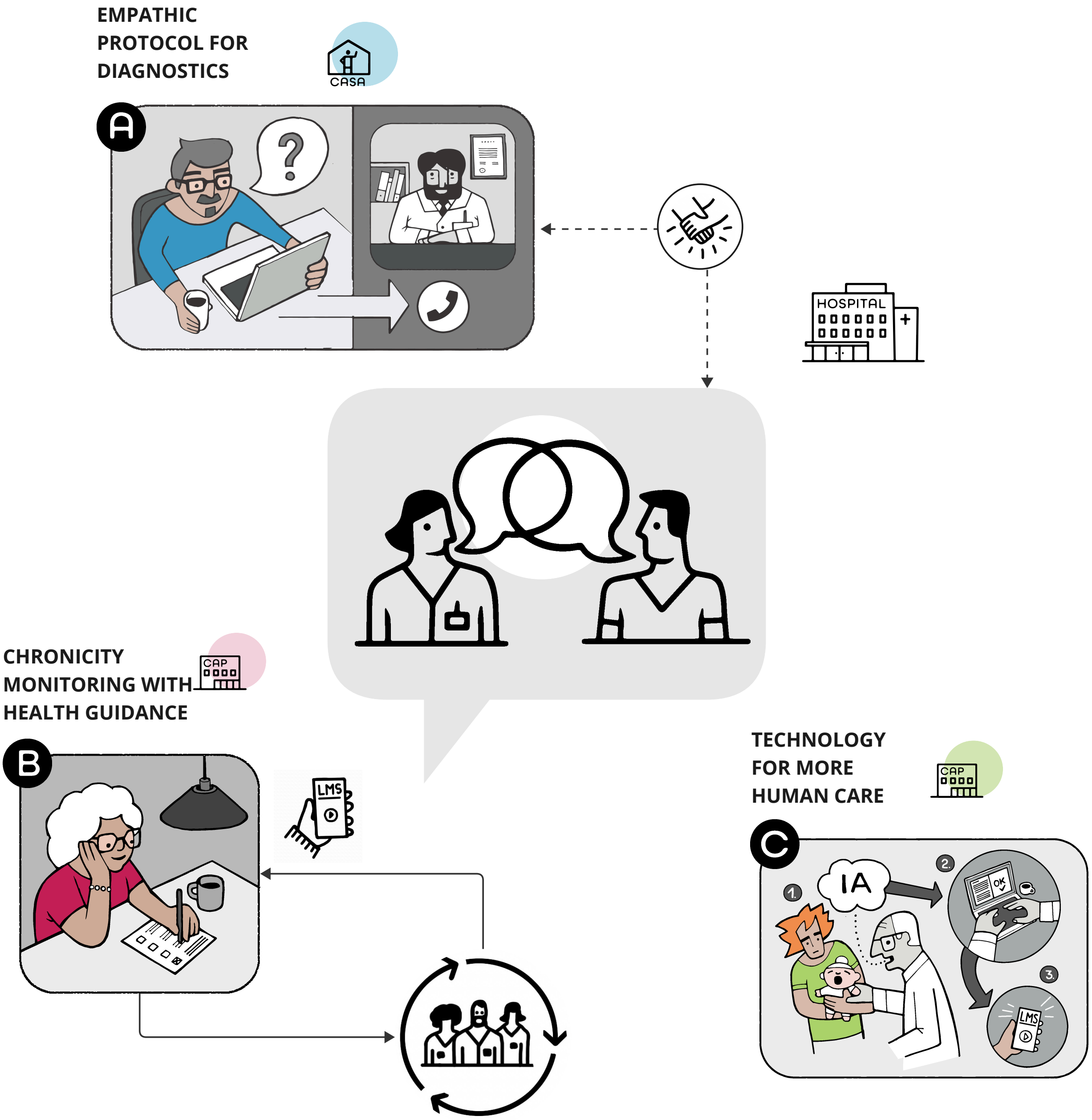

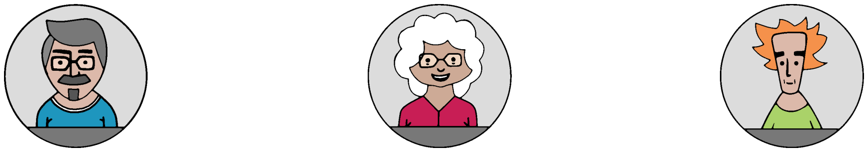

**RAMON,** 55 years	**DOLORS,** 68 years	**MIQUEL,** 34 years
Solsona	Amposta	Olot

A future scenario in which professional-patient interactions transform into more human and empathetic conversations thanks to new follow-up roles, new protocols for complicated conversations, and technological support that frees up time to listen, connect, and provide personalized and close care.

COLLABORATIVE AND CONTINUITY CARE

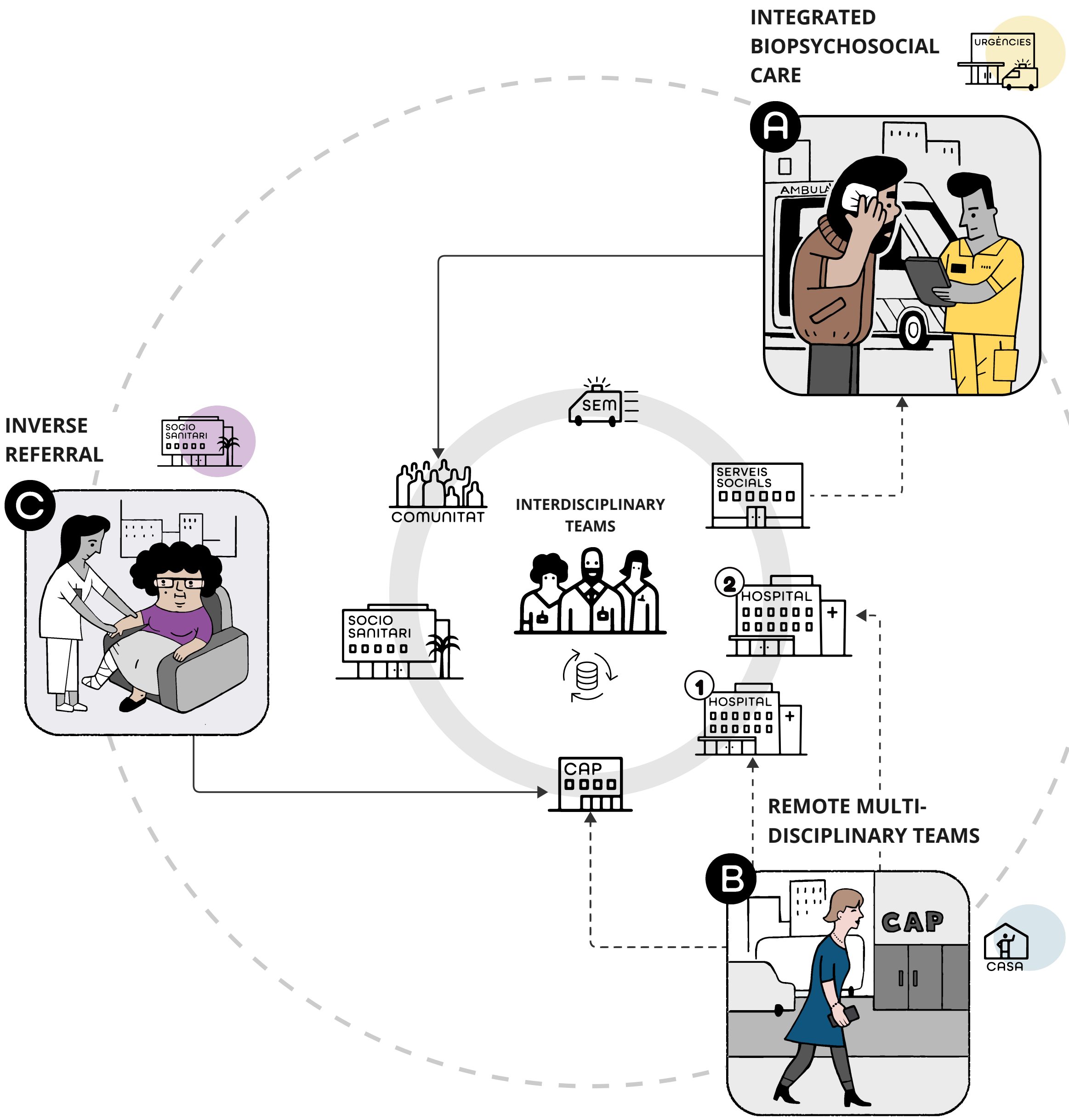

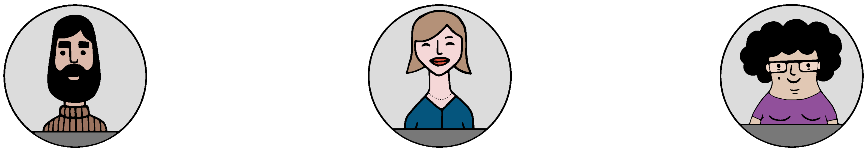

CARLES, 37 years

Barcelona

NADIA, 21 years

Reus

MARIA, 67 years

Terrassa

A future scenario in which collaboration and coordination between lines and levels of care is a reality, and where referral to specialized care is always accompanied by a return referral to local care, the space where a multidisciplinary team centralizes the continuous monitoring of the person's needs.

PERSONALIZED AND PROACTIVE HEALTH PREVENTION AND PROMOTION

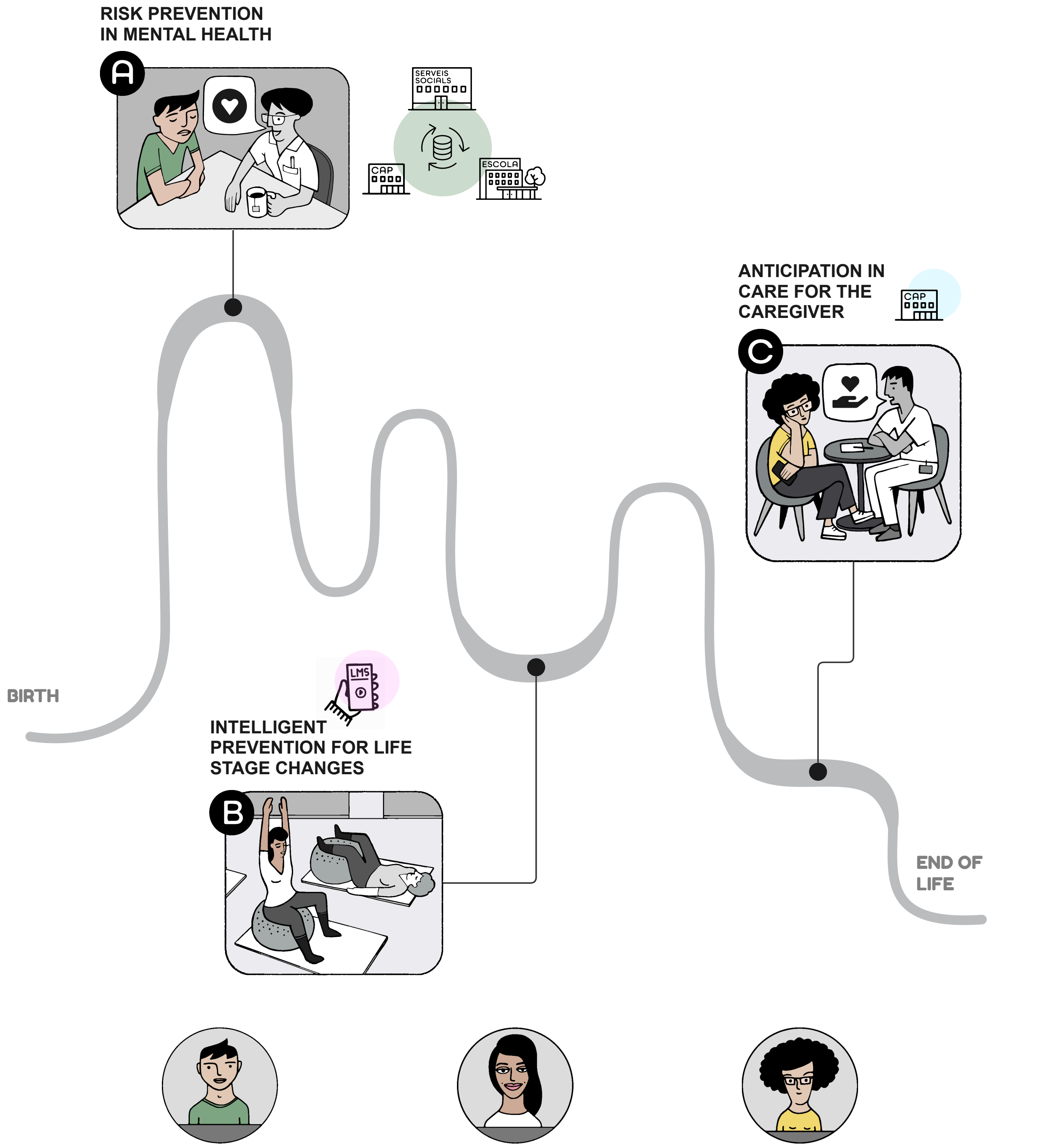

ARNAU, 17 years

Lleida

FLORA, 49 years

Manresa

ROSA, 40 years

Sabadell

A future scenario in which health promotion and prevention are designed according to the specific needs of each population group and life stage, and care is personalized with a proactive system that integrates advanced predictive models to identify and respond dynamically to potential health risks before they manifest.

ACTIVE PARTICIPATION IN HEALTH

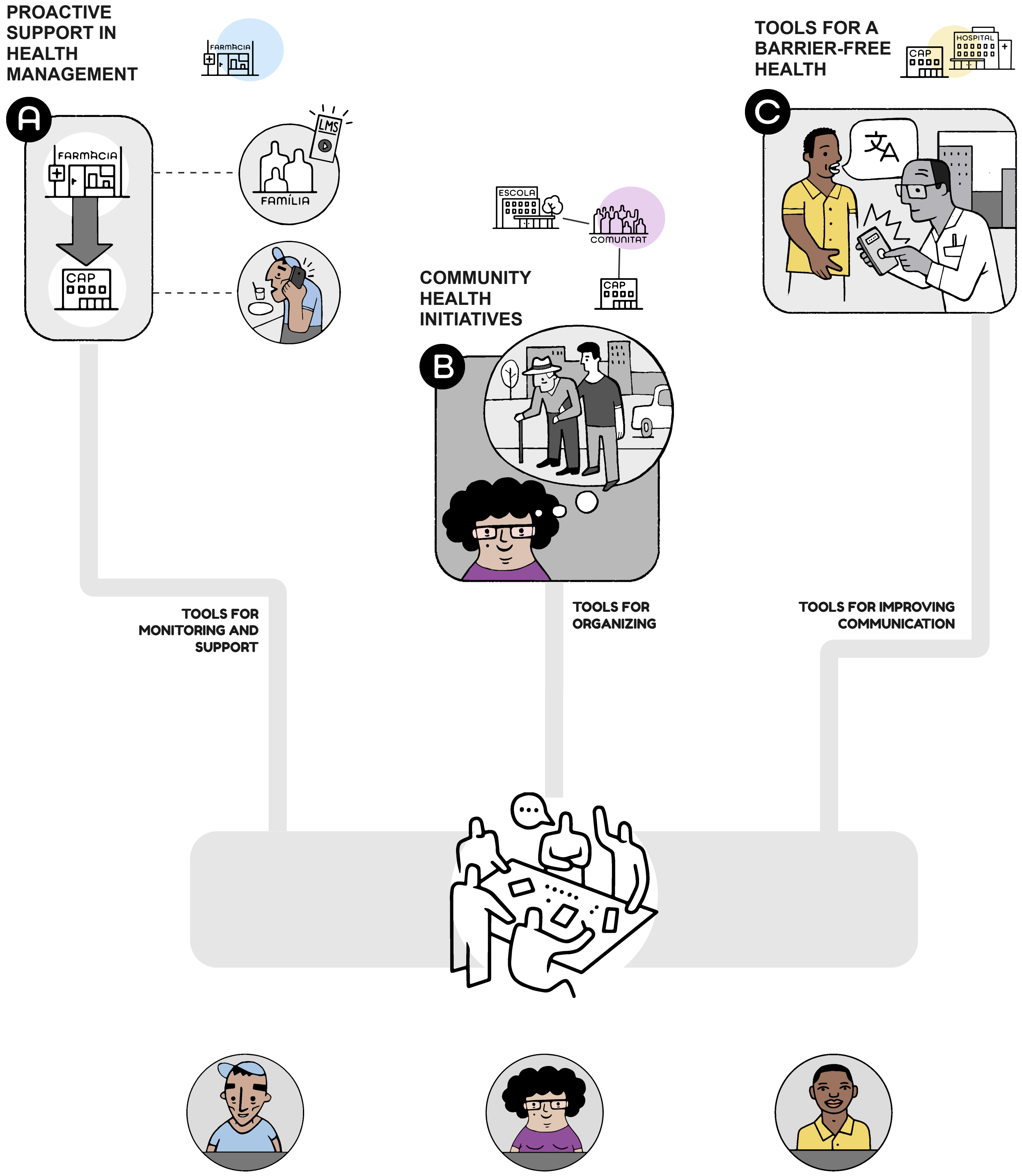

JOAN, 72 years

Vilafranca del Penedès

MARIA, 67 years

Terrassa

MUSA, 26 years

Vic

A future scenario in which health is built as a shared responsibility, with empowered individuals making shared decisions, and informed thanks to education, support, active participation, and the power to choose care models.

HOLISTIC VIEW OF HEALTH

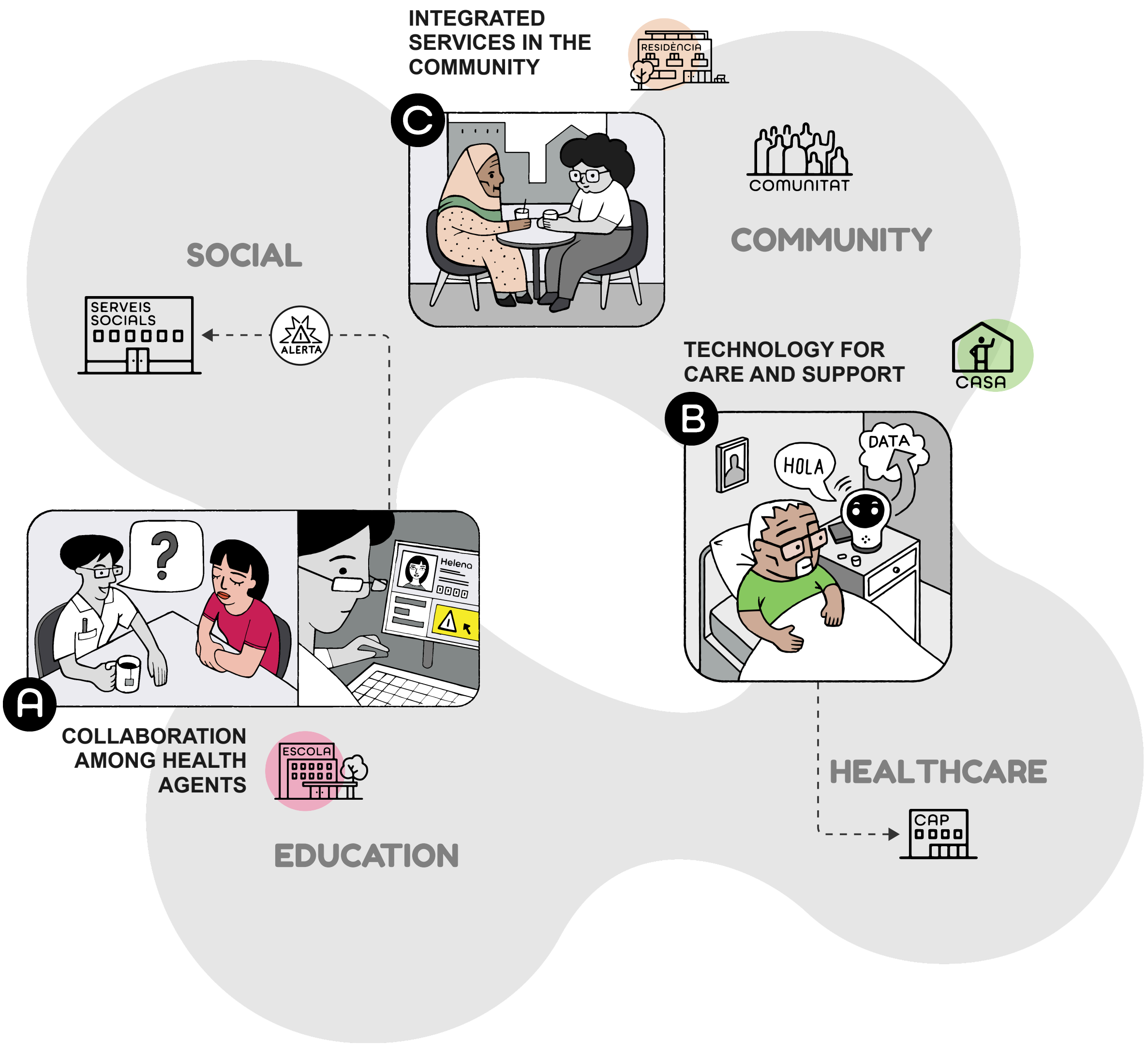

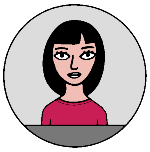

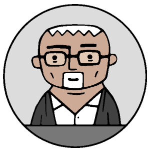

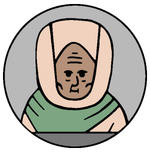

HELENA, 14 years
Móra d'Ebre

PEPE, 65 years
Palamós

FÀTIMA, 90 years
Igualada

A future scenario in which health is managed from an ecosystem perspective, integrating the social, educational, health, and community spheres, with a cross-cutting culture based on multidisciplinary and diverse teams, which include health specialists as well as community actors, and that offer comprehensive care and support centered on the person.

Collective vision for the evolution to the future care model

COLLECTIVE VISION FOR THE EVOLUTION TO THE FUTURE CARE MODEL

Current healthcare model

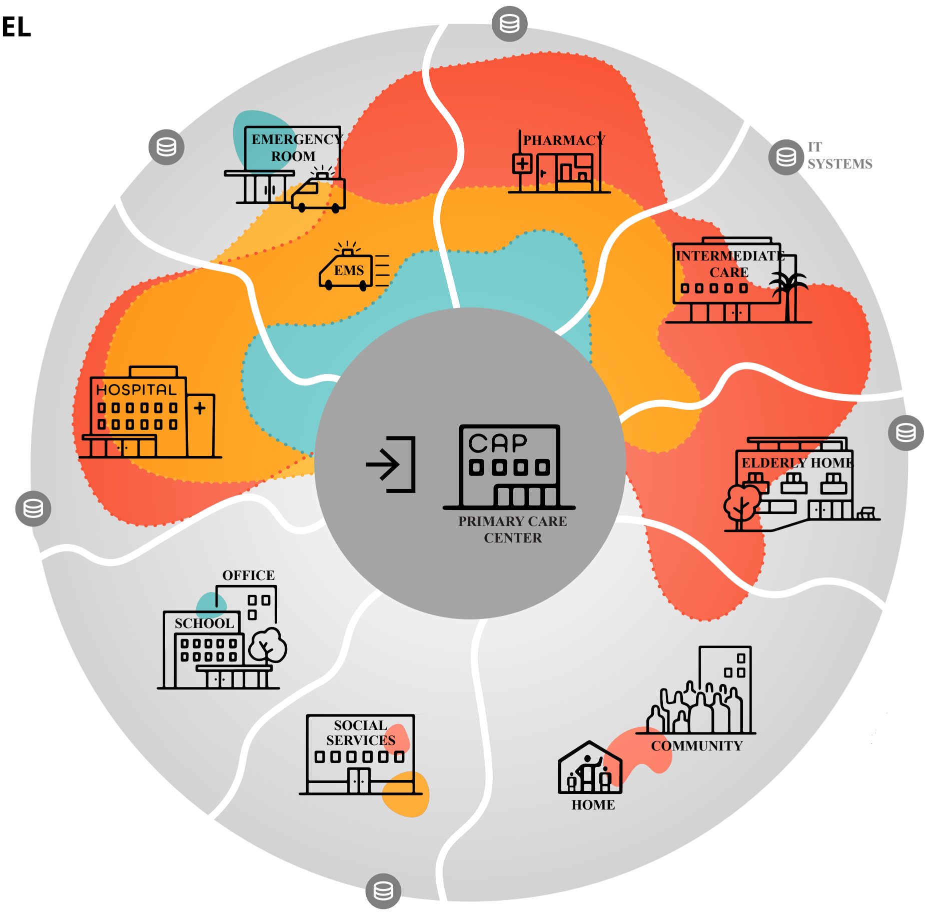

Future care model

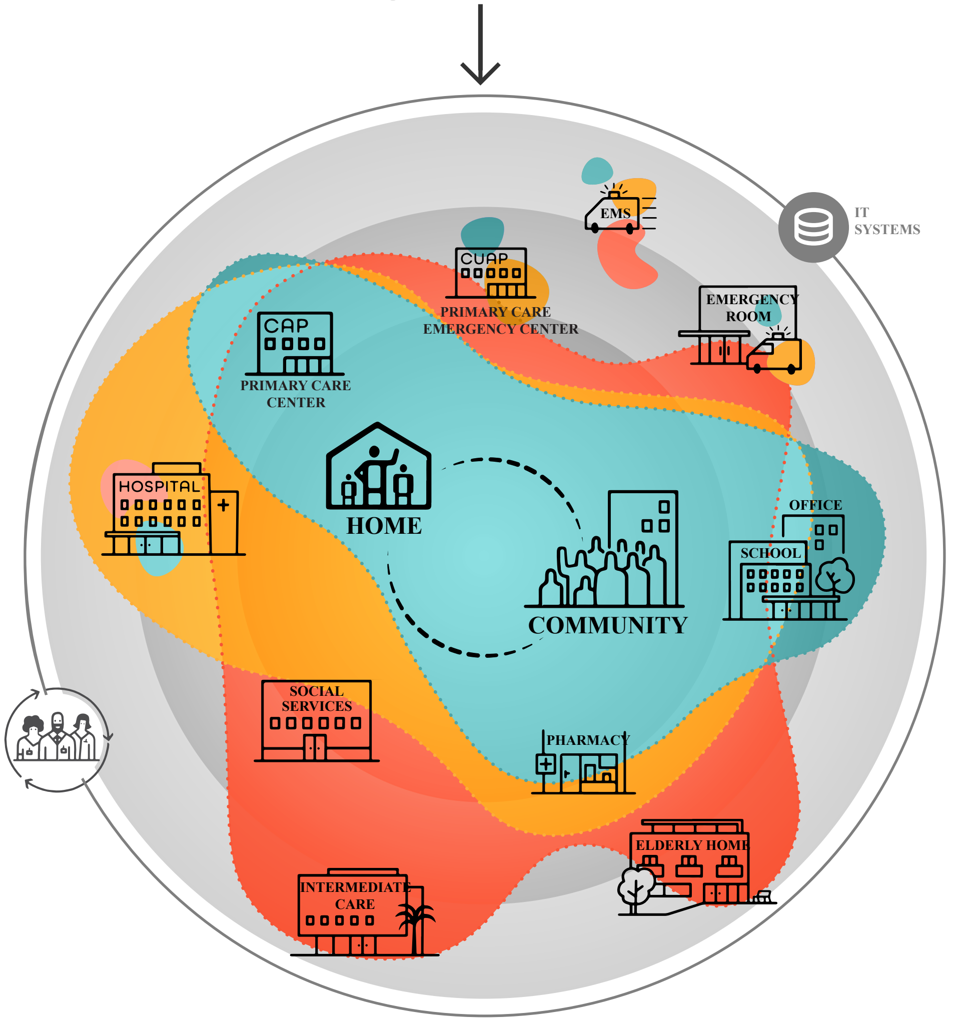

- Not at-risk or non-vulnerable population
- Population at-risk
- Population with complex health needs

Participation survey – citizens & caregivers

We invite you to evaluate your experience during today's session

Thank you very much for taking part in today's session. We truly appreciate your time and contributions.

We would like to ask for your opinion regarding the session you attended. Your feedback helps us to continue improving our mission of listening to and gathering the voices of patients and families, and of facilitating their participation in service improvement processes. If you were accompanied, each of you may respond separately.

1. What did you like most about the session

2. What would you have liked to do differently? How can we improve these sessions?

3. How would you rate the following aspects?

	Very positive	Positive	Neutral	Negative	Very negative
Open and safe space to share experiences and ideas					
Facilitation and workshop dynamics					
Respectful interactions					
Overall value of the workshop and information gathered					
Opportunity to connect with other individuals with similar experiences					

Finançat per la Unió Europea NextGenerationEU · Plan de Recuperación, Transformación y Resiliencia · Pla de Recuperació, Transformació i Resiliència · /Salut · Next Generation Catalunya · Generalitat de Catalunya

4. Do you feel you were given the support and information needed to participate easily and effectively? (please select one option)

- ▢ Yes
- ▢ No
- ▢ Not sure

5. Do you feel that your views and experiences were listened to and taken into account? (please select one option)

- ▢ Yes
- ▢ No
- ▢ Not sure

6. Do you have any additional comments or feedback you would like to share with us?

Thank you very much for helping us improve this experience!

Finançat per la Unió Europea NextGenerationEU

Pla de Recuperació, Transformació i Resiliència

/Salut

Next Generation Catalunya

Generalitat de Catalunya

Participation survey – professionals

We invite you to evaluate your experience during today's session

Thank you very much for participating in today's session. We really appreciate your time and contributions.

We would like to know about your experience regarding the methodology of the workshop you attended. This will help us understand what worked well and what didn't, so we can improve the experience and dynamics of future workshops.

1. What did you like most about the session

2. What would you have liked to do differently? How can we improve these sessions?

3. Did you receive enough information to participate in or prepare for the workshop? Would you have liked to receive any additional materials or preparation? (please select one option)

☐ Yes, the information provided was sufficient

☐ No, I did not receive any information

☐ I would have appreciated receiving additional materials or preparation

☐ Please specify which ones:

Finançat per la Unió Europea NextGenerationEU
Pla de Recuperació, Transformació i Resiliència
/Salut
Next Generation Catalunya
Generalitat de Catalunya

4. Do you feel that your views and experiences were listened to and taken into account? (please select one option)

▢ Yes
▢ No
▢ Not sure

5. How would you rate the following aspects?

	Very positive	Positive	Neutral	Negative	Very negative
Open and safe space to share experiences and ideas					
Facilitation and workshop dynamics					
Respectful interactions					
Overall value of the workshop and information gathered					
Opportunity to connect with other individuals with similar experiences					

5. What do you think about the duration of the workshop? (select one option)”

☐ It was too long
☐ It was adequate
☐ I would have liked to stay a bit longer

6. Do you have any additional comments or feedback you would like to share with us?

Thank you very much for helping us improve this experience!

Finançat per la Unió Europea NextGenerationEU
Pla de Recuperació, Transformació i Resiliència
/Salut
Next Generation Catalunya
Generalitat de Catalunya
