# Supplementary material for: Cocreating Principles for Digital Health Equity: Cross-Sectional, Qualitative Study for Participatory Human-Centered Design in Catalonia
Source: J Med Internet Res. 2026 Jan 6;28:e84129. doi: 10.2196/84129 (PMC12774400; doi:10.2196/84129)
Supplement: Multimedia Appendix 2 [file jmir-v28-e84129-s002.pdf]

01 | The disconnection between the health and social systems makes it difficult to provide comprehensive and holistic care

BARRIER

Facing the growing number of people with **complex needs**, we face the challenge of offering more **holistic** care through an **effective integration** of health with different areas, especially **social care**. Although there is widespread agreement on the importance of this integration and there are already promising initiatives in some territories, practice still reveals many **barriers**. Beyond the need for common **governance, funding and management**, differences in the **culture, approach and objectives** of each system hinder the co-creation of unified services.

Having to apply for my mother's dependency law was hell... First with the Social Worker at the CAP, then she sent the report to the City Council and now after 9 months we are still waiting for the decision. I need help, I can't take it anymore!
CAREGIVER

Social problems end up falling on us: we do everything, request aid, find them transport...
HEALTH PROFESSIONAL

Everything to do with the social system is a black box. I don't know what happens there. And we should know! 80% of health determinants are social.
HEALTH MANAGER

80%

of the determinants of health are found **outside the health system** [8]

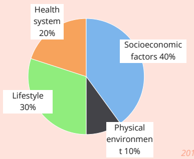

+130.000

dependency assessment requests handled annually [13]

2024

74.000

dependent people on the waiting list to receive aid or a review of the diagnosis [14]

2024

➼ Integrated social and health care is one of the objectives of the Department of Health for the XV legislature [2]. Putting the person at the center of their health and of the system, reducing fragmentation of the care process and promoting collaboration between public actors are priorities of the Catalonia Health Plan 2021-2025 [3]. The integrated care model is one of the guiding principles of the 30 measures to strengthen the health system from the Committee of Experts for the Transformation of the Public Health System [5].

CONSEQUENCE

PERSON RECEIVED AND CAREGIVER

- **Lives firsthand** the intersection between the health and social problems they suffer, and feels **abandoned** by the system for not being able to address them jointly as needed, with the Dependency Law being one of the most representative cases.

HEALTH PROFESSIONAL

- Lacks information about the **context** of the people they care for, such as their social determinants or care networks, to be able to offer better **treatment and follow-up**, as well as to identify the additional support they may need from institutions.
- The lack of channels and protocols between systems **makes it impossible** to **report** the problems observed, for example during a home visit.

HEALTH MANAGER

- Due to the **inefficiencies** and lack of coordination between the social and health systems, as well as situations where some social problems end up causing health problems, it is difficult to **anticipate and prevent** correctly admissions to the health system and contribute to its decongestion.

01 | The disconnection between the health and social systems makes it difficult to provide comprehensive and holistic care

OPPORTUNITY

How might we...

- How might we address health from the **whole set of a person's dimensions** and not only from healthcare, to promote well-being with a complete and balanced perspective?

- Establish a **coordinated** information system between the social and health sectors that allows professionals to carry out a **multidimensional assessment** of patients' needs?

If we want to move forward, we need good connections between departments, with those we relate to: education, social services, justice...
HEALTHCARE MANAGER

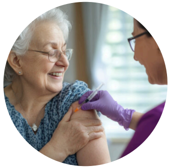

Salut + Social Viewer

The government of the Generalitat de Catalunya has interconnected social and health information systems, allowing professionals to access users' dependency and health data. Currently, more than 498 professionals use the Salut + Social viewer, which will expand to 109 basic social services. [35]

CATALONIA

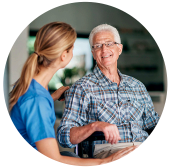

Integrated Home Care

The Integrated Care Services of Aran are promoting a project that seeks to improve data management and care for the population of the Vall d'Aran by integrating social and health models, using artificial intelligence to anticipate problems and personalize care. [36]

VALL D'ARAN

02 | It is increasingly necessary to offer support for emotional well-being with a preventive, holistic and community approach

BARRIER

In the face of the current mental health crisis, there is a significant increase in needs related to people's **emotional wellbeing**, which makes it essential to adopt a broader, more **holistic and community-based** health approach than the current healthcare system offers. However, Mental Health and Addictions Care has **limited resources** that are primarily allocated to specialized care for mental disorders, resulting in a lack of promotion of emotional wellbeing and **effective prevention** at the **community and educational** level.

> *Here the networks are not working. There are many associations and they work very well, but the primary care centers and hospitals do not know about them.*
>
> *The associations are extremely important, because they care for the patient and the caregiver.*
> **PERSON RECEIVING CARE**
>
> *We are medicalizing social problems. People seek medical and individualized solutions to issues that go beyond that.*
> **HEALTH PROFESSIONAL**
>
> *Mental disorder is not the same as distress. More public education is needed.*
> **HEALTH MANAGER**

In 2021, **39% of girls** and **20% of boys** aged

13-19 years

showed **emotional distress** [10]

2023

It is estimated that the **pandemic** has increased the cases of

28% major depression

26% anxiety disorder

worldwide [15]

2024

These are **determinants** of mental health [10]:

- Unwanted loneliness
- Lack of social support
- Job insecurity
- Domestic and caregiving work
- Poverty
- Lack of sleep

2023

According to the World Health Organization

1 in 4 people

will experience some **mental health problem** during their lifetime [5]

2020

➼ Mental health is one of the objectives of the Department of Health for the XV legislature, focusing on infants, children and young people [2]. Mental health and emotional wellbeing is one of the priorities in the health objectives of the Catalonia Health Plan 2021-2025, with numerous action plans [3].

CONSEQUENCE

PERSON RECEIVING CARE AND CAREGIVER

- It faces a public system lacking the resources to respond to current emotional wellbeing and mental health needs. This often makes mental health seem like a **low priority**.
- People look for spaces to be **heard and emotionally supported**, which they find outside the public healthcare system, often in patient associations.

HEALTHCARE PROFESSIONAL

- Social problems that are not addressed preventively in the community end up falling on the healthcare system and **increase the care burden** on professionals.
- They feel they do not have the **adequate resources or tools**, and they experience difficulties connecting with community resources to carry out their work.

HEALTHCARE MANAGER

- It considers that Mental Health and the health system bear the burden of responding to problems that do not have a health origin, but are **social problems that end up being medicalized**.

02 | It is increasingly necessary to offer emotional well-being support with a preventive, holistic and community-based approach

OPPORTUNITY

How might we...

- Involve the **community** in promoting emotional well-being so that the person feels they have **accessible accompaniment and preventive support**?

- Deploy technologies that enable an **effective follow-up** of users' physical health, mental health and emotional well-being **over time**, including all involved stakeholders?

Community and neighborhood networks are great health prescribers and could be protective and informative elements.
HEALTHCARE MANAGER

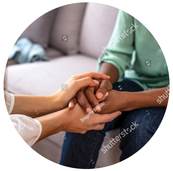

PCP at eCAP

The Collaboration Program between Mental Health and Addictions and Primary and Community Care is an established project that offers services and activities to improve mental health care. It is implemented through the joint work of primary and community care teams and mental health and addictions teams. [37,38]

CATALONIA

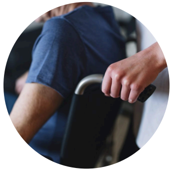

Caregiver school or programs

In Catalonia, several programs offer training and support to informal caregivers, aiming to improve the quality of care and caregivers' well-being. The programs include training, emotional accompaniment and practical support for people who care for family members or loved ones. [39,40,41]

Expert Caregiver Program

SJD Caregivers School

Emotional Support and Mutual Aid Groups

03 | The new expectations and demands of citizens towards health services pose additional challenges for the system

BARRIER

Society is changing and new generations present very different **values, expectations and demands** regarding the **experience of the care** they receive. They prioritize **, flexibility in care and having fast and direct communication channels with health professionals. This, if not properly managed, together with the increase in everyday social problems that are often ended up being handled by the health system, creates greater pressure on professionals and the health system.**

I don't understand going to the CAP and not being given an appointment until the afternoon or the next day, when you see it is empty.
PERSON RECEIVING CARE

A patient has a face-to-face visit, a telephone call, a scheduled appointment, eConsulta... They can connect through many places. If the patient has a scheduled visit they should not be able to request another one. There are expert users who have several and saturate the system.
HEALTH PROFESSIONAL

The message about the importance of mental health is given and this makes people feel they should have a regular psychologist, but this is not viable; the system cannot sustain it.
HEALTH MANAGER

2.766.646
Inquiries and alerts received at **061** during 2023 [12].
2023

4.434.881
appointments scheduled **digitally** in 2023 [12].
2023

➸ Digital transformation is one of the objectives of the Department of Health for the XV legislature [2] and one of the guiding principles of the 30 measures to strengthen the health system from the Committee of Experts for the Transformation of the Public Health System [5].

CONSEQUENCE

PERSON RECEIVING CARE AND CAREGIVER

- Feels **unease** when they do not receive a quick response or lack direct communication channels with the professional and do not know how to manage their situation.
- The **weakening of family and community networks to provide care support** means that on some occasions the health system is the only support in the face of an illness.

HEALTHCARE PROFESSIONAL

- Experiences **digital noise** from the use of multiple open channels of communication with patients and among professionals, often for **issues beyond the medical field.**
- Feels frustrated for **not being able to provide the care they would like** due to the growing attention to demands and urgencies.

HEALTHCARE MANAGER

- Difficulty in **responding to the new expectations** of citizens regarding speed and reasons for consultation, because the system is not prepared to respond to this volume of demands.

03 | The new expectations and demands of citizens towards health services pose additional challenges for the system

OPPORTUNITY

How might we...

- Adapt care services to meet expectations of **proximity**, **immediacy, and flexibility**, while making citizens share responsibility?

- Implement digital communication channels that facilitate direct interaction between patients and health professionals, improving flexibility and immediacy in care and management?

E-consultation saves me many visits to the primary care center, because you write to the family doctor and they renew your medication.
PERSON RECEIVING CARE

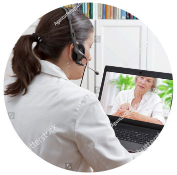

Teledermatology

Coordination with primary care for the early diagnosis of skin cancer. Images of suspicious lesions are shared via ECAP with dermatology, which diagnoses within 24/48 h and refers to urgent surgery. This has doubled or tripled diagnoses, improving the patient experience and the quality of care. [42,43]

VILADECANS

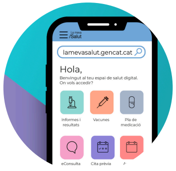

Non-Face-to-Face Care

Catalonia currently has non-face-to-face care (ANP) channels such as telephone care, eConsulta and video consultation. In the case of eConsulta, this asynchronous messaging service between primary care professionals and citizens has already reached one million users. [44]

CATALONIA

04 | A population more educated in health and prevention would be more self-sufficient and would make more efficient use of the health system

BARRIER

Health education is key to improving self-care, preventing diseases and ensuring the sustainability of the health system. An informed person **makes better decisions**, **manages their illnesses better** and **avoids unnecessary consultations**. Despite advances in health promotion and shared decision-making, the **lack of time and capacity** limits both professionals and organizations. In addition, the **low integration with the community environment** reduces opportunities to foster a more self-sufficient population in managing their health.

You feel somewhat guilty because you don't know if you are doing it well enough. There is a lack of training to know what we can and cannot do.
CARE PERSON

Some patients cannot assess the real urgency of their situation. A father went into the emergency room for his child's otitis because they did not want to wait for the visit to the primary care center in the afternoon.
HEALTH PROFESSIONAL

It cannot all fall on healthcare... We need health to be taught from schools and the community.

There is great difficulty in making users understand the purpose and use intended for each resource. Citizens must be educated; people go to emergency rooms because they don't know where to go.
HEALTH MANAGER

50%

of the hospital emergencies attended by the ICS **during 2023** could have been resolved in a primary care center [26]

2023

➸ Promoting citizens' commitment to community health, through health education and the promotion of self-care, is one of the 30 measures to strengthen the health system from the Committee of Experts for the Transformation of the Public Health System [5].

CONSEQUENCE

PERSON RECEIVING CARE AND CAREGIVER

- Both the person cared for and the caregiver have a **feeling of insecurity** due to the lack of information on how to manage the treatment of some illnesses from home.
- Turning to unofficial or unverified sources for information can pose a **risk** to their health.

HEALTH PROFESSIONAL

- Feels frustrated by the increase in **consultations considered unnecessary**, such as a headache, which prevent them from dedicating that time to other patients or more essential tasks.
- They **lack connection with the community fabric**, which makes it difficult for this network to help better inform the person being cared for.

HEALTH MANAGER

- Detects an increase in avoidable consultations that strain the system and considers that these resources could be allocated to good **education** of the population.
- Experiences greater management difficulty due to the strain on some care lines, especially emergencies, for having to allocate resources to cover the need for an **immediate response**.

04 | A population better educated in health and prevention would be more self-sufficient and would make more efficient use of the health system

OPPORTUNITY

How might we...

- Integrate **health education and prevention** into people's daily lives to enhance their autonomy and **shared responsibility** in managing their health and using the system?

- integrate information technologies that provide **educational resources** on self-care and prevention to the population, as well as connect with resources from the **community network**?

> *Could there be a digital resource to explain to citizens when to use one service or another. For example, it could indicate when I should go to the primary care center (CAP) or when I should use the emergency services.*
> ***HEALTH PROFESSIONAL***

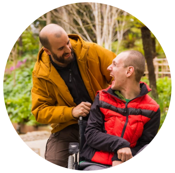

Catalan School of Health

The Catalan School of Health is a space to acquire skills and knowledge to maintain, enhance and improve health, to make informed decisions about it and increase well-being. The School offers a selection of content, tools and resources in health education. [45]

CATALONIA

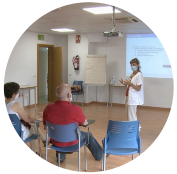

Aula Salut

Aula Salut Vilafranca is a collaborative space between Social Services and Primary Care to train caregivers over 6 months. It includes practical sessions, such as the effective use of LMS, to improve care for dependent people at home. [46]

VILAFRANCA

05 | Social, language and digital barriers make it difficult for many people to access the health system and use it correctly

BARRIER

Social, digital and language gaps **condition access** to the health system. Excessive use of **medical terminology** and **language** barriers make comprehension difficult for many people. Added to this are difficulties in using **digital tools**, especially among older people, which limit their ability to manage their health. These barriers must be addressed to ensure **equitable care** and that everyone can manage their health and navigate a system that is already complex.

Many times I don't understand the doctors' calls. If they give me a biopsy result I remain the same, or I have to say, please tell me in words I understand. I prefer to go to the place than to receive a call.
PERSON RECEIVING CARE

It is very hard to move through the current health system, especially when you have to knock on many doors, particularly when you are old. Either you are inside the system or you are dead. Caregivers deserve an award.

There are patients, especially the older ones, who confuse a virtual appointment with an in-person one and who do not read SMS messages and then miss appointments and we have to reschedule them.
HEALTH PROFESSIONAL

48%

of the **foreign population** is at risk of **poverty or social exclusion** [18]

2024

➸ Putting the person at the center of their health and the system and adapting resources to guarantee equity and quality of care are priorities of the Catalonia Health Plan 2021-2025 [3]. The #HealthWithoutHome working group has defined 23 proposals to improve access to and healthcare for people experiencing homelessness in Catalonia [19].

CONSEQUENCE

PERSON RECEIVING CARE AND CAREGIVER

- They do not understand **medical language** and feel defenseless and insecure. This endangers their ability to follow **medical instructions** correctly.
- In the case of a language gap, they are not **autonomous** when interacting with the health system without a person or translation mechanism.
- In the case of a **digital** gap, they feel lost, as they do not understand how to contact the doctor or why things "are not done as before".

HEALTH PROFESSIONAL

- They are forced to allocate **additional time and resources** to ensure understanding, either acting as a translator, spending more time than planned giving explanations, or teaching non-digitalized patients how to use tools like *La Meva Salut*.

05 | Social, language, and digital barriers prevent many people from accessing the health system and using it correctly

OPPORTUNITY

How might we...

- Ensure that the tools and services of the healthcare system are **accessible and understandable** for everyone, regardless of their social, language, or digital **context**?
- Design digital health platforms that are intuitive and easy to use for people with **different levels of digital, socio-cognitive, or language skills**, making it easier for professionals to communicate more effectively?

The healthcare system must account for the budget to make health services accessible to everyone via technology.

We need to be able to move forward toward a single-window system. It will make life easier for both the user and us.

HEALTH MANAGER

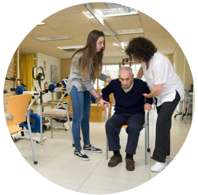

Student volunteering

In Palamós, an initiative has been launched where secondary school students volunteer to help older people download and use applications like the LMS. This project not only fosters intergenerational solidarity but also helps improve digital autonomy. [47]

PALAMÓS

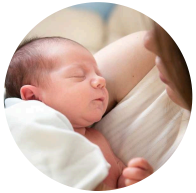

Espai Nadó

Espai Nadó is a service for new families, offering comprehensive support, resources to overcome language barriers, and a welcoming place to share experiences and activities with other families and professionals. [48]

GIRONA

06 | More resources are needed to address the growing needs of an aging population with higher levels of frailty and complexity

BARRIER

The **aging** of the population and the increase of patients with **frailties, chronic conditions and comorbidities** highlight the lack of adequate support for **older people**, especially in nursing homes. This also affects **caregivers**, who demand more tools, spaces for rest and support to manage their emotional burden. Despite advances in care for complex cases, a **more solid infrastructure** of professionals and facilities is needed to care for both the people receiving care and their caregivers.

I started caring for my mother, I live with her and it's 24 hours. I don't know where to go; no one explains to me how to manage it as a caregiver.

The public nursing home is already a dream. My mother, because she has level 2 dependency and receives an allowance, can afford it.
CAREGIVER

Nursing homes have become centers with advanced chronic pathology and dementias. Training and role specialization are needed to address this growing need.

It looks great to offer services for young people, but care for the elderly is left behind.
HEALTH PROFESSIONAL

...
HEALTH MANAGER

62%
It is estimated that this will be the increase of the Catalan population **over 65 years** compared to 2024. [28]
2024

15.8%
of people over 65 had a **recognized dependency** in 2023 [16]
2024

Nursing homes will need to **increase the number of professionals** by
20,000
to face the aging of the "baby boomers" [17]
2024

The year 2023, 19% of **those over 65 years old** were over 84 years old. By **2050**, it is estimated that the % says of [16]
24.3%
2024

➸ Healthy and active aging is one of the objectives of the Department of Health for the XV legislature [2]. The Department of Health and the Department of Social Rights are already working on the Deployment Plan for integrated social and health care for people living in nursing homes (RGG) in Catalonia [6].

CONSEQUENCE

PERSON RECEIVING CARE AND CAREGIVER

- In situations of economic vulnerability, they often suffer **undesired loneliness** for not being able to access a nursing home or for the lack of a support network.
- The dependent person **cannot receive the care they require** nor the support to be able to die with dignity and according to their preferences.
- Caregivers, for their part, **feel guilty** for the lack of time or knowledge, and experience distress due to difficulties managing the **dependency law**.
- Although day centers and intermediate care offer relief, access is complicated without resorting to the private sector.

HEALTH PROFESSIONAL

- They encounter increasingly **medically complex cases** and lack resources to respond.
- Those working in nursing homes feel **questioned**. They also have worse working conditions than in healthcare and lack **incentives** to want to work there.

HEALTH MANAGER

- With the increase in loneliness, they see how intermediate care ends up **functioning as containment**, due to the lack of response capacity of nursing homes.
- They think the system of care and attention for aging needs to be **completely rethought**.

06 | More resources are needed to address the growing needs of an aging population with higher levels of frailty and complexity

OPPORTUNITY

How might we...

- Provide aging care that guarantees **personal preferences** and responds to the growing needs of the **aging population** and **caregivers**?
- Create an information system that integrates the needs of both frail older people and their caregivers, facilitating access to available services and resources?

Resources should be moved to the patient, instead of moving the patient to the resources. For example, remove care homes from the equation and move toward a system that prioritizes home care, or turn a shared apartment for older people into a care residence.

HEALTH MANAGER

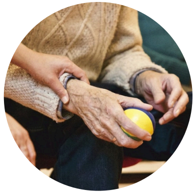

School of Caring

The School of Caring is a project that offers support to non-professional caregivers who care for people with dementia or disabilities. It is a joint initiative of the Amposta Primary Care Centre (CAP), aiming to improve caregivers' quality of life through training and emotional support. [49]

AMPOSTA

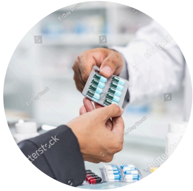

Atenea, AI

Aimed at older people in Lleida, the ATENEA project consists of a voice assistant, which is a tablet without buttons or touch screens, that reminds them daily if they have taken their prescribed medication. The assistant asks several times and if there is no response an SMS is automatically sent to a family member. [50,51]

LLEIDA

07 | Waiting lists affect people both emotionally and in access to services, and can reflect other problems in the system

BARRIER

Long **waiting lists** entail health risks and a strong **emotional impact** due to the **uncertainty** in diagnoses and treatments, affecting both the people receiving care and their caregivers. They also hinder and limit the development of **work and personal life**. Despite the positive assessment of **public and universal healthcare**, because of the consequences of waiting to receive the necessary test or treatment, many people are forced to turn to **private healthcare**, generating **inequalities in access** to care.

For a year and a half I was drowning and they only gave me antihistamines; since they didn't take you seriously I went to the private sector to get tests and some inhalers

I am an electrician, I am self-employed, and they gave me an appointment for physiotherapy after 8 months. I can't wait, how will I eat? If you don't have private care, you're stuck.
PERSON RECEIVING CARE

When patients go to private care, then I have to interpret what they tell me because I can't consult it in their record. Sometimes they tell me they were operated on for things that aren't operable.
HEALTHCARE PROFESSIONAL

64%
of patients take more than 5 days to access the **GP** [7]
2024

Waiting lists of
1 to 3 years
for **residences** with **public** places [11]
2024

Average waiting time of
75 days
for routine **psychology** appointments [27]
2024

Waiting time of
150 days
for a **surgical intervention** [7]
2024

➻ The Department of Health proposes reducing the waiting time to 48 hours to receive care in Catalan primary care. "When 48 hours are exceeded we already have the person in a hospital emergency department," Minister Pané has stated [7].

CONSEQUENCE

PERSON RECEIVING CARE AND CAREGIVER

- Suffers **multifactorial anxiety** derived from the **uncertainty** about the progression of the illness, and the feeling of **not having control** over their time or the practical aspects of their life.
- Besides worsening the illness, waiting can hinder **returning to work**, with the risk of losing their job and causing other negative consequences in the social and working life of the people receiving care.
- Faces the **decision** or the **impossibility** of having to assume costly private services.

HEALTHCARE PROFESSIONAL

- **Feels the helplessness** of having to care for frustrated people who are in a **worse state** for having endured waiting lists.
- Does not have access to **updated medical data** of those people who have accessed private healthcare services.

07 | Waiting lists affect people both emotionally and in access to services, and can reflect other problems in the system

OPPORTUNITY

How might we...

- How might we **reduce the impact** of waiting lists on people's health and emotional well-being by offering **tailored information** so they can better manage their daily lives while they wait?

- establish a **prioritization system** that uses clinical data to identify patients with more urgent needs and speed up waiting times?

A way should be found for the patient to authorize sharing data from tests and procedures done privately with the public health system.

HEALTHCARE PROFESSIONAL

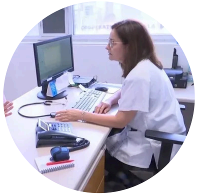

Scheduling by reason

To improve the organization of primary care, a pilot plan has been carried out in 14 CAPs to schedule visits using a computer program that focuses on the reasons users request them. The objective is to require the best possible treatment in each case and the professional in charge. [52]

CATALONIA

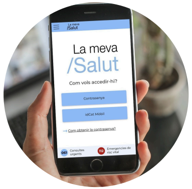

LaMevaSalut Information

La Meva Salut informs users on the surgical waiting list about waiting times, the status of the request and the center's average, ensuring transparency and proper management of expectations. [53]

CATALONIA

08 | The use of quantitative and non-health criteria in planning and allocating resources creates inefficiencies in the system

BARRIER

We are moving toward a **value-based** model, using tools like PROMs and PREMs. However, the **planning of resources and financing** of the health system is still focused today on **quantitative criteria**, such as the number of interventions performed, rather than on the **real care needs** of patients. On the other hand, the absence of a territorially adapted strategy causes **regional inequalities** in facilities, services and professionals, with a direct impact on the quality of care received by the population.

El Pallars is a tourist and mountain county. It makes no sense that in summer and winter staff is reduced and we don't have traumatologists when that is when there are more high-risk sports.
PERSON RECEIVING CARE

They pay you for discharges, for visits, for interventions; everything is based on boxes. That does not help to work according to the needs and preferences of the person receiving care.
HEALTHCARE PROFESSIONAL

We are not looking at the future of what the citizenry will be like, we have migratory movements that change the population. We should adapt planning to all that.

We talk a lot about focusing on the patient, but we treat them according to how each area is funded.
HEALTH MANAGER

€1,781.86

expenditure by SCS per **inhabitant** in 2023 [12]

2023

14,077.55 M€

expense recorded by the SCS in 2023 [12]

2023

Distribution of SCS expenditure [12]

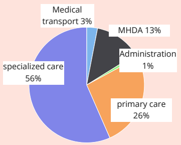

MHDA: Hospital medication dispensed on an outpatient basis

2023

➼ Evidence and evaluation is one of the objectives of the Department of Health for the XV legislature [2]. Adjusting resources is a priority of the Catalonia Health Plan 2021-2025 [3]. Sufficient financing and modernization of center management are guiding pillars of the 30 measures to strengthen the health system from the Committee of Experts for the Transformation of the Public Health System [5].

CONSEQUENCE

PERSON RECEIVING CARE AND CAREGIVER

- In some cases the person does not receive the care they need but the care that is available according to the distribution of resources.
- When the system does not adapt sufficiently to territorial particularities, a situation of **inequity** is generated towards the person receiving care and the caregiver, who find that they cannot access the care they need close to home, and are forced to **travel**.

HEALTH PROFESSIONAL

- Does not receive the support to care for the patient according to their needs, since they receive incentives to **meet metrics** that do not align with **health outcomes.**

HEALTH MANAGER

- Must **prioritize covering expenses**, which forces facilities to adapt their resources to what is funded, and not to the real care needs.
- To incorporate new roles, they need to collect indicators that justify their suitability in order to hire more similar profiles.
- When adapting centralized guidelines to the specific needs of the territory they **find themselves limited**.
- Acts according to current needs, but **lacks data to plan** based on changing needs.

OPPORTUNITY

How might we...

- Prioritize **health outcomes** and an **equitable distribution** of resources so that people feel they receive fair, local care regardless of where they live?

- Develop an information system that integrates data to assess the real impact of health interventions on patients' health, the effectiveness of public health interventions, and adjust resources accordingly?

As a professional, you make the diagnosis, but when it comes to implementing it sometimes you can't because a specialist is missing. Someone at a higher level should map specialists so we can zoom in and see, for example, the difference between supply and demand in areas like allergies and dermatology.

HEALTHCARE PROFESSIONAL

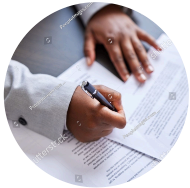

Payment for Results

CatSalut has introduced a contracting model based on Payment for Results (PPR), which combines fixed and variable payments. This mechanism is aimed at achieving high standards in health outcomes, clinical efficiency, patient experience and coordination among providers. [54,55]

CATALONIA

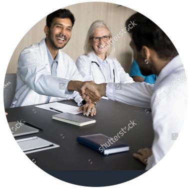

AQuAS Guide

Practical guide for professionals and healthcare managers who want to drive changes in de-implementation of low-value practices, shared decision making and care improvement. It offers clear steps, strategies, actions and tools to promote a cultural change in health organizations. [56,57]

CATALONIA

09 | A highly fragmented and specialized system hinders the role of primary care as a connector of services for coherent care

BARRIER

There is a **wide portfolio of services** based on a multidisciplinary vision, accompanying different **life moments or processes**, and that are highly valued by people receiving care. Even so, they operate in a fragmented way due to **hyper-specialization**, generating multiple referrals that break care continuity. **Primary and Community Care** has the potential to act as a connector role between different care services, but it is under great **pressure** from demand for care, lack of funding and the growing bureaucratic burden on healthcare professionals.

I do miss having my usual primary care doctor who is always my doctor. Everything is referred to emergencies. That leaves you feeling alone.
PERSON RECEIVING CARE

PADES is excellent, he was able to die at home and it was my salvation, I couldn't have done it alone.
CAREGIVER

Primary care must be the real entry point and place of stay for care. It should not be a place of referral. Good work must be done and primary care should be strengthened more.
HEALTHCARE PROFESSIONAL

People have needs; if we section ourselves, the more little boxes we make, the worse for the patient, because needs are not isolated.
HEALTH MANAGER

630,000
more in-person visits to Primary Care in 2023 than in 2022 [32]
2024

2,252,671
referrals to specialists in 2023 [12]
2023

61%
of **emergency consultations** in 2023 could have been handled by Primary Care, according to the Catalan Health System Results Hub [33]
2023

The **expenditure on Primary Care** has been
lower in 2023 than in 2022
both in absolute terms (-€36M) and relative terms (-1.5%) [32]
2023

➼ Strengthening Primary and Community Care is one of the objectives of the Department of Health for the XV legislature [2]. Reducing fragmentation of the care process and promoting collaboration among public actors are priorities of the Catalonia Health Plan 2021-2025 [3]. Granting primary care teams autonomy in managing human and economic resources is one of the 30 measures to strengthen the health system from the Committee of Experts for the Transformation of the Public Health System [5].

CONSEQUENCE

PERSON RECEIVING CARE AND CARE-GIVER

- In cases of comorbidities that require multiple specialists, it must assume the **responsibility for coordination** of referrals, visits and tests. This entails risks for errors and inequalities, especially in vulnerable people or those with less capacity to assume this role.
- May be forced to travel between **different points of care** without having sufficient information.
- Feels **neglected** in delicate and uncertain moments.

HEALTH PROFESSIONAL

- Fragmentation and hyper-specialization across multiple services are perceived as a sign of the weakness of Primary Care.
- They must spend time referring to specialized services, and then experience difficulties consulting results and conducting continuous follow-up of the people cared for.

HEALTH MANAGER

- They must face inequalities in care due to the availability of services in each Health Region and the providers operating there.

09 | A highly fragmented and specialized system hinders the role of primary care as a connector of services for coherent care

OPPORTUNITY

How might we...

- Rethink the organization of specialized support and the coordination between services at different levels so that the person perceives a **close**, continuous and uninterrupted care?

- Develop a digital platform that integrates all care services, facilitating communication and coordination among health professionals to improve continuity of care?

If Primary Care is to be the central axis of the system's collaboration, it needs more recognition and to increase its problem-solving capacity.
HEALTH MANAGER

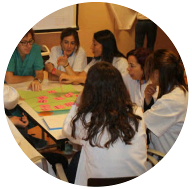

UBA3+

UBA3+ are primary care units made up of a stable team of a doctor, a nurse and an administrative clinical reference with advanced competencies. This organizational model improves coordination and patient follow-up, especially for those with chronic diseases, thanks to personalized care. [58]

CATALONIA

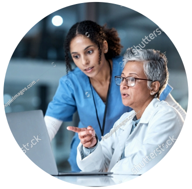

Bidirectional communication

System being tested in Osona for bidirectional communication between primary and hospital care. It allows scheduling appointments, managing tasks and sending queries between doctors and nurses, improving patient coordination and follow-up. [59]

OSONA

10 | Protocols of action and resources must be strengthened for daily support of chronic conditions and mental illnesses

BARRIER

The health system stands out in cases of **urgency and critical care**, thanks to protocols that facilitate decision-making to address these situations quickly. However, there is a lack of reinforcement in **continuous and preventive care**, and adequate support for people in the **daily management** of chronic diseases, revealing shortcomings in community and local support. This also hinders the prevention of decompensations, which lead to **avoidable** hospitalizations. In the case of mental illnesses, the lack of support is worsened by the scarcity of resources and the associated stigma.

Mental health has been left to God; more resources should be allocated to it.

People don't make that click like they do with cancer; there isn't that bond. With mental health, people turn their faces away.
SERVICE USER

When I began caring for my mother, besides her character, I live with her and it's 24 hours. I don't know where to go; no one tells me anything about how to manage myself as a caregiver.
CAREGIVER

In moments of emergency and maximum criticality we have very good and powerful hospitals, and we have it very proceduralized and clearer.

Mental pathology "pure and simple" (schizophrenias, eating disorders...) is "the great forgotten". It works well in critical moments and acute hospitalization, but it doesn't have enough resources for chronic, continuous, community-based care.

37.8%

of the adult population aged 15 and over suffers from a **chronic disease** [3]

2024

Since 2017 there has been a **percentage increase** of

9.84%

chronic patients in Mental Health [31]

2023

Funds raised by **La Marató de TV3** as an indicator of the visibility of diseases in society [70]

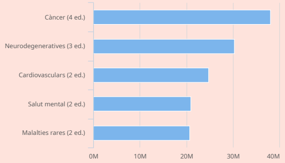

➸ Ensuring home care for people in situations of dependency and chronicity, coordinated by primary care and other health and social resources, enhancing the role of family and community nursing is one of the 30 measures to strengthen the health system from the Committee of Experts for the Transformation of the Public Health System [5].

CONSEQUENCE

PERSON RECEIVING CARE AND CARE-GIVER

- They lack orientation and social, emotional and practical support to manage the day-to-day of the illness; which can lead to the worsening of their condition.
- In cases of mental health and cognitive decline, families feel helpless and without support to know how to make decisions and manage difficult situations.
- Both patient and caregiver end up seeking support on their own, especially from patient associations.
- Inequalities and inequity are generated in the care of the population, since some conditions with high public awareness and more resources have established follow-up protocols and support (such as some oncological processes), versus other conditions with a very high stigma, both from society and professionals, that do not have adequate support (e.g. a person with schizophrenia without psychological support).

HEALTH PROFESSIONAL

- Has **difficulties in properly following up** with the people served, since other aspects such as the number of visits made or conducting an initial visit are incentivized more than maintaining treatment.
- From Primary Care, there are difficulties in following up patients coming from hospital care, due to the **lack of a reverse referral** and access to information.

10 | Protocols of action and resources must be strengthened for daily support of chronic conditions and mental illnesses

OPPORTUNITY

How might we...

- Improve the **monitoring** of chronic diseases so that the person feels **constant, preventive and tailored support** to their day-to-day needs, regardless of their pathology?

- Develop a digital platform that integrates continuous and preventive care resources, facilitating access to information and support for daily management of chronic conditions?

The initiative "Care School" is a great thing for us; they come a couple of times a month and explain how to do rehabilitation exercises.
CARE-GIVER

There should be more reverse referrals. When someone is discharged from the hospital, they should be transferred to primary care.
HEALTH PROFESSIONAL

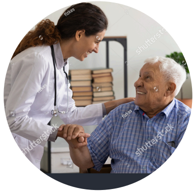

Home care Mental Health

At the Parc Taulí Health Corporation the Home Hospitalization Unit in Mental Health was implemented with the aim of improving care for patients with severe mental disorders who require intensive and acute care for psychopathological decompensations. [69]

SABADELL

11 | Various factors contribute to the burnout of health professionals, a situation that, in turn, negatively impacts the quality of care they can provide

BARRIER

The **high turnover of healthcare professionals** is a phenomenon that significantly affects the quality and continuity of care. It is due both to the **work culture** and changing values of new generations of professionals, who often seek more work-life balance, and to the **stressful working conditions** that characterize the health sector. Although many people receiving care highly value the attention they receive and the drive and enthusiasm of professionals, turnover hinders the creation of **bonds of trust and empathy**, essential for effective care and which require time to build.

My psychologist has changed every so often. Every time you go you have to expose yourself again and explain what is happening to you.

My doctor retired and was replaced by a pregnant woman who did one visit and disappeared. Another came, was given a grant, and she also disappeared.
PERSON RECEIVING CARE

I have even thought about leaving medicine and changing jobs. I work a ton of hours and there is no help anywhere, everyone does their own thing. I am taking diazepam for anxiety.

I'm not only talking about salary, but about this feeling of being recognized and, in quotes, respected. Both by patients and by the institution.
HEALTHCARE PROFESSIONAL

We train residents, but soon they leave for other services, quit the profession or even leave the country. They finish residency and request another specialty, or even say: "I'm not cut out for this".
HEALTHCARE MANAGER

247%

and the increase in **medical leave for mental health** among doctors between 2022 and 2023 [24]

2024

Hiring in the health sector [25]

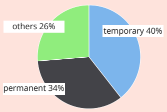

Global hiring across all sectors [25]

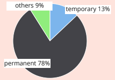

2024

➼ Professionals: the core of the health system is one of the objectives of the Department of Health for the XV legislature [2]. Health professionals as the foundation of the system is a priority of the Catalonia Health Plan 2021-2025 [3]. Professional leadership is one of the inspiring axes of the 30 measures to strengthen the health system from the Committee of Experts for the Transformation of the Public Health System [5].

CONSEQUENCE

CARE RECIPIENT AND CAREGIVER

- High turnover in primary care leads to the loss of information about their **personal and family context**, which comes from mutual familiarity over time. This perception is more pronounced in older people, who internalized the "Family Doctor" model, as well as in long-term conditions.
- In more sensitive cases, such as mental health, they feel **exposed** every time they have to repeat their health story.

HEALTHCARE PROFESSIONAL

- Poor working conditions and difficulties in work-life balance reduce their **motivation** and encourage job changes.
- Lack of job stability **prevents them from forming bonds** with people receiving care.
- They must **constantly adapt** to new environments and systems. Turnover increases the risk of losing **critical information**.

HEALTHCARE MANAGER

- The **lack of stable teams** affects work dynamics and forces investment of time and resources in **hiring and training** staff, often from other countries, who need to adapt to the local context.
- The **rigidity of the system** makes it difficult to integrate new ways of working that could improve professionals' wellbeing and motivation.

11 | Several factors contribute to healthcare professionals' burnout, a situation that in turn negatively impacts the quality of care they can provide

OPPORTUNITY

How might we...

- Ensure **cohesive care** with a **smooth and complete transfer of information** between professionals, so that patients perceive that their **context is well known** and can establish **bonds of trust**, even in situations of staff rotation?
- Design digital tools that facilitate the collection of patient information before consultations, allowing doctors to devote more time to active listening and personal interaction during visits?

Attention should go beyond a single person. It could be a reference team instead of a reference doctor.

HEALTH MANAGER

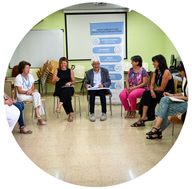

Meeting between different providers

In Osona meetings are organized between healthcare and social providers to foster collaboration and plan joint actions. The goal is to improve integrated care, optimize resources and share information about how different entities work. [60,61]

OSONA

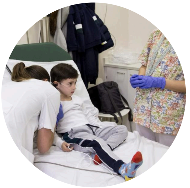

SJD pediatrics agreement

The agreement between SJD and the Berga Hospital allows professionals to travel once a week to provide service, improving care in the area. This benefits both Berga and SJD and offers specialists experience in various settings. [62]

BARCELONA

BERGA

12 | The administrative burden in daily management makes it difficult for healthcare professionals to provide optimal care

BARRIER

The **increase in bureaucratic burden** and the use of multiple communication channels in the daily work of healthcare professionals negatively impact their ability to provide **quality and empathetic** care. The health system, designed with a more **procedural** than caregiving approach, increases the number of bureaucratic tasks and hinders coordination between services. Although the **administrative** professional already alleviates this burden through triage, there is a lack of clarification and professionalization of the role.

Now they almost make you take a test to diagnose a simple cold. The doctor no longer listens with a stethoscope, and there is always a computer in the way. This multiplies the need for resources for tests.

They didn't treat my father's tongue cancer in time because the specialist had to spend a lot of time doing paperwork.

PERSON RECEIVING CARE

I spend more time looking for the patient's information than attending to them, and while I search for a patient's lab result someone suddenly calls me to ask about something else. You can't work like this!

We have become information managers. We spend 4 minutes entering information and dedicating time to information and 1 minute dedicating time to the patient.

HEALTHCARE PROFESSIONAL

159

doctors per 100,000 inhabitants in 2023. Downward trend over the last 20 years [21]

2023

In Spain, health professionals spend

30-40%

of their time on **administrative tasks** [22]

2023

10 min.

This is the **average duration of a visit** with the doctor in Primary Care [23]

2017

The number of **prescriptions per person has increased**

6.45%

between 2021 and 2023 [30]

2023

➸ Adapting resources to ensure equity and quality of care is a priority of the Catalonia Health Plan 2021-2025 [3]. Promoting innovation in the health system, with new organizational and technological models, is one of the 30 measures to strengthen the health system proposed by the Committee of Experts for the Transformation of the Public Health System [5].

CONSEQUENCE

CARE RECIPIENT AND CAREGIVER

- They perceive a more **distant** treatment when they feel healthcare professionals focus their attention on entering data into the system. Some people, especially older adults, miss the doctor's visit as they used to know it: with a conversation and use of the stethoscope instead of the computer.
- Intermediary filtering figures, such as administrative staff, create the need to share context and sensitive information, generating a sense of distrust and **lack of privacy.**

HEALTH PROFESSIONAL

- They experience more **wear and tear**, feeling that increasingly more evidence is needed to justify diagnoses and that the level of bureaucracy for each step is excessive.
- The collection of information falls on the professional, who spends more time managing information and responding to emails than **attending the patient** or studying the cases that come to them.

HEALTHCARE MANAGER

- The **over-bureaucratic and rigid system** makes it difficult to propose deep structural changes, so solutions to try to reduce bureaucracy focus more on **liaison roles or the use of technology** and artificial intelligence.

12 | The administrative burden in daily management makes it difficult for healthcare professionals to provide optimal care

OPPORTUNITY

How might we...

- Streamline administrative processes to **free up time and resources** from professionals' daily work so they can focus more on person-centered care?

- Implement an information system that enables health professionals to better manage their workloads, reducing stress and improving their job satisfaction?

> *There should be an automatic process that allows you to spend more time with the patient. Here some technology like an AI or a different way of entering information could play an important role.*
>
> **HEALTH MANAGER**

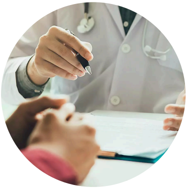

AI for the medical record

Six CAPs will pilot the AI tool Relisten, which transcribes conversations between doctors and patients to extract relevant information and facilitate the medical record. This technology reduces administrative tasks by up to 20%, allowing for more humane care and improving the work environment.[63]

CATALONIA

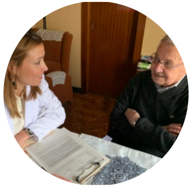

Local healthcare administrative

The CAP Ramon Turró in Barcelona has implemented the "Local healthcare administrative" project, which consists of home visits by healthcare administrative staff to facilitate procedures for patients with mobility difficulties, thus improving access to the healthcare system.[64]

BARCELONA

13 | The multiplicity of information systems hinders effective coordination and collaboration among professionals, necessary for continuity of care

BARRIER

The current technological infrastructure is based on **multiple information systems** with limited interoperability that hinders **fluid collaboration and coordination** between different levels of care and system actors. The **Shared Clinical Record** is seen as a solution with great potential by healthcare professionals, as it facilitates access to relevant information. However, access to this information is not **agile** nor complete, and there is a lack of **homogeneous guidelines** for its sharing.

When I moved from Vilafranca to Palamós, they lost my medical record.
PERSON RECEIVING CARE

The feeling is one of chaos and poor organization. A friend's husband was lost and they didn't even know in which hospital he was.
CAREGIVER

I have to delete emails to be able to receive new ones, and I can't keep a history. It's dangerous to work like this, we lose patient information and make decisions without knowing their history well.
HEALTHCARE PROFESSIONAL

Providers work with different programs. The information system is very fragmented and leads to duplications.
HEALTHCARE MANAGER

In the group of **69 acute hospitals** there are

29

different information systems owned by different companies [20]

Projection of

+16,000

pieces of information related to patients, with EMRs being the most important source [20]

➸ Digital transformation and innovation is one of the objectives of the Department of Health for the XV legislature [2]. Promoting innovation in the health system, with new organizational and technological models, accelerating the digital transformation of the health system to improve efficiency and accessibility, as well as implementing a system to tailor prescriptions to the person and their situational diagnosis are among the 30 measures to strengthen the health system proposed by the Committee of Experts for the Transformation of the Public Health System [5].

CONSEQUENCE

CARE RECIPIENT AND CAREGIVER

- They have to **repeat information** and provide context when it is not recorded in the medical history, or **travel multiple times** for the same test that needs to be repeated.
- The need to repeat tests or the lack of information can cause possible errors in diagnosis and treatment, and **compromise their safety.**

HEALTHCARE PROFESSIONAL

- The lack of **access** to the person's context and preferences makes **decision-making** about treatments and interventions difficult and entails **inefficiencies and delays**, such as an increase in consultations between professionals or multiple referrals.
- They spend a lot of **time** entering and consulting information in systems that are slow and obsolete.

HEALTHCARE MANAGER

- The **inefficient use of** human resources and equipment, triggered by the lack of information sharing, impacts the ability to manage well.
- Not having common guidelines that define which **criteria** to follow when uploading information means the decision falls on each organization, causing the HC3 information to be incomplete.

13 | The multiplicity of information systems hinders effective coordination and collaboration among professionals, necessary for continuity of care

OPPORTUNITY

How might we...

- Improve **access and the flow of information** between healthcare professionals, so that the person perceives that the system **knows their situation well** and does not feel forced to **repeat** information?

- Establish **uniform protocols and guidelines** of interoperability for the sharing of data between different levels of care and information systems, ensuring that relevant information is available when needed?

The information should be accessible to all professionals because the information belongs to the patient, not to the professionals.
HEALTHCARE PROFESSIONAL

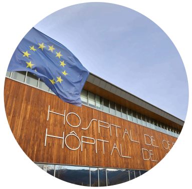

Border Puigcerdà Hospital

It works in a network with health and social-health services, regardless of whether they are in the French state or in Catalonia. The purpose of the AECT HC is the construction of a cross-border hospital intended to care for patients from the territory of Cerdanya (Spain) and Capcir (France). [65,66]

CERDANYA

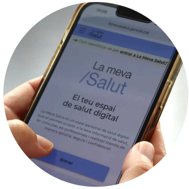

Blue Button

Catalonia is a pioneer in the EU with the *Blue Button*, a feature of *La Meva Salut* that allows users to share their clinical data securely and with interoperability according to European standards. Thus enabling data sharing between public and private healthcare. [67,68]

CATALONIA
